# Comprehensive taxonomy and worldwide trends in pharmaceutical policies in relation to country income status

**DOI:** 10.1186/s12913-017-2304-2

**Published:** 2017-05-25

**Authors:** N. Maniadakis, G. Kourlaba, J. Shen, A. Holtorf

**Affiliations:** 1 Department of Health Services Organization, National School of Public Health, 196 Alexandras Avenue, 115 21 Athens, Greece; 2 EVROSTON LP, Athens, Greece; 3 Collaborative Center of Clinical Epidemiology and Outcomes Research (CLEO), Non-Profit Company, Athens, Greece; 4 Head Market Access, Abbott Products Operations, Hegenheimermattweg 127, AG 4123 Allschwil, Switzerland; 5 Managing Director, Health Outcomes Strategies, Colmarestrasse 58, 4055 Basel, Switzerland

**Keywords:** Pharmaceutical/drug policy, Pharmaceutical/drug pricing & reimbursement, Health technology assessment, Pharmaceutical/drug demand controls

## Abstract

**Background:**

Rapidly evolving socioeconomic and technological trends make it challenging to improve access, effectiveness and efficiency in the use of pharmaceuticals. This paper identifies and systematically classifies the prevailing pharmaceutical policies worldwide in relation to a country’s income status.

**Methods:**

A literature search was undertaken to identify and taxonomize prevailing policies worldwide. Countries that apply those policies and those that do not were then grouped by income status.

**Results:**

Pharmaceutical policies are linked to a country’s socioeconomics. Developed countries have universal coverage and control pharmaceuticals with external and internal price referencing systems, and indirect price–cost controls; they carry out health technology assessments and demand utilization controls. Price-volume and risk-sharing agreements are also evolving. Developing countries are underperforming in terms of coverage and they rely mostly on restrictive state controls to regulate prices and expenditure.

**Conclusions:**

There are significant disparities worldwide in the access to pharmaceuticals, their use, and the reimbursement of costs. The challenge in high-income countries is to maintain access to care whilst dealing with trends in technology and aging. Essential drugs should be available to all; however, many low- and middle-income countries still provide most of their population with only poor access to medicines. As economies grow, there should be greater investment in pharmaceutical care, looking to the policies of high-income countries to increase efficiency. Pharmaceutical companies could also develop special access schemes with low prices to facilitate coverage in low-income countries.

**Electronic supplementary material:**

The online version of this article (doi:10.1186/s12913-017-2304-2) contains supplementary material, which is available to authorized users.

## Relevant Topics for decision makers

- The present study aims to provide a worldwide empirical characterization of pharmaceutical policies and systems
- This map includes an analysis of policies from 33 European countries (including Turkey), 8 Middle Eastern and African countries, 9 American countries (including the USA), and 13 countries from the Asia Pacific region
- The taxonomy and categorization help to understand the breadth of policies applied to regulate pharmaceutical markets
- The categorization approach can be utilized to identify best practice policy mixes per health system to improve system efficiency in terms of cost and outcomes.
- This study may be used as a foundation for an analysis of the impact of such policies.

## Background

Modern healthcare systems aspire to provide accessible, effective and efficient healthcare services for all individuals living in any given county. Attaining these objectives is increasingly challenging in the context of an environment with financial constraints, increasing demand due to better coverage, rising expectations and population aging, and rapidly emerging new technologies alongside scientific advances that impact the organization and cost of delivering care [1, 2]. Another reason for the increasing challenges facing health authorities and payers is the continuous increase in the price of new technologies, and specifically medicines. An example of the latter is the prices of cancer drugs, which have risen by up to tenfold in recent years, as well as those for orphan diseases; commentators are now worried that if this trend continues it could potentially bankrupt healthcare systems, given the number of new cancer medicines in development in association with prevalence trends [3–5].

Moreover, there is currently a great deal of inefficiency in healthcare, due to the size, diversity and complexity of healthcare systems on the one hand and market failure factors on the other [6, 7]. Inefficiencies also exist because of the considerable pressures put on healthcare systems by various stakeholders (patients, industry, prescribers, politicians) to fund new technologies—often at high prices despite their limited health gain—diverting funding from a more efficient use [8–11]. In addition, countries have often been found to pay too much for generics, especially now that many standard medicines available in this form, with prices of generic medicines and biosimilars varying by up to 30-fold between different countries [12, 13].

In this context, it becomes increasingly difficult to create conditions that will guarantee the long-term sustainability of healthcare systems. Healthcare delivery in many countries is heavily regulated by governments with the aim of assuring equitable access to needed services whilst controlling expenditure and increasing efficiency. The degree of regulation and intervention has increased in recent years, following the recent economic crisis [14–16]. Often, much of the attention and policies are focused on pharmaceuticals, as they represent an important component of healthcare provision. Pharmaceuticals contribute significantly to the effectiveness and outcomes of health care delivery, and hence to human wellbeing, on the one hand, but on the other, they also consume a significant proportion of healthcare expenditure [16, 17]. Pharmaceutical expenditure in high-income countries absorbs up to 30% of total healthcare expenditure [18], whilst in low- and middle-income economies it may be much higher than that, accounting for up to 60% of total expenditure. In this context, as most of the expenditure is in the form of out-of-pocket payments, illness can be catastrophic for families [19].

Regulations and policy interventions may have a significant impact on the operation of a pharmaceutical market. Good examples come from countries such as Sweden and the Republic of Ireland. Demand side measures in Sweden appreciably increased prescribing of generic (multiple sourced products) versus patented products. The reverse was seen in Ireland with very little demand side to combat industry promotion [20–22]. Finding the right balance is a challenging endeavor. A completely unregulated pharmaceutical market may prove suboptimal from the perspective of societal wellbeing, whilst on the other hand severe regulation may also lead to inefficiencies in certain settings and may have detrimental effects in the medium and long term [23–29].

Typical pharmaceutical policies intervene on the demand as well as the supply side of the market, mainly to control their pricing, reimbursement, prescription, and dispensing, as well as the quality of care in relation to proper drug use and the overall cost of pharmaceuticals [30–32]. A variety of different approaches may be adopted to regulate each of the aforementioned domains, and choosing between them poses significant considerations. Moreover, it is rather challenging to combine them in a meaningful manner and to develop a right mix of policies, which will optimize access, effectiveness and the efficiency of pharmaceutical use given the available resources. There is no standard universal solution to this challenge and each case is different, as the prevailing characteristics of the healthcare, social, political, and economic environment all play a significant role. Hence, there is considerable interest in this field and much research has been undertaken to provide insights into how different policies in relation to existing or new therapies are meeting objectives in pharmaceutical markets [24, 30, 33–38].

The aim of this paper was to review and classify the pharmaceutical policies utilized worldwide and to identify prevailing patterns across countries at different levels of economic development. Its contribution stems from the fact that it focuses on the entire policy spectrum, not just a single policy dimension, and that it takes a global view. The countries reviewed represent more than 80% of the global population and close to the entire global pharmaceutical expenditure, while reflecting vastly divergent economic, societal and health care settings.

## Methods

A literature search was undertaken using internet search engines such as Google, electronic article databases such as Medline and Embase, and the web sites of relevant organizations such as the WHO (World Health Organization) and ÖBIG (Österreichisches Bundesinstitut für Gesundheitswesen), the OECD, the European Commission and ISPOR. The search keywords used are presented in the three groups of Table 1. For each country in the first group, the policy terms in the second group were combined with the pharmaceutical identifiers in the third. The literature review, up to March 2014, identified 2978 published articles, conference presentations, and reports. These were all then reviewed for inclusion by two independent members of the research team (NM, GK) and were subsequently classified. A total of 870 publications in English were reviewed further and 562 were selected for inclusion in the study, either because they described the pharmaceutical care in a single country or multiple countries (311), or because they were relevant methodological papers focusing on the subject matter of this present study (251). They were no strict inclusion/exclusion criteria as this was not a systematic review. To be considered, studies needed mainly either to describe the pharmaceutical landscape in one or many countries, or to discuss or review methodological and policy aspects. The countries evaluated were 63 in total; they are listed in Additional file 1, alongside the related literature sources used for each of them. This effort took considerable time; however, given the nature of the study, the fact that more recent publications were not considered is not likely to have had an impact.

**Table 1:** Terms used in the literature search

Group	Terms
Countries	*Europe:* Austria, Belgium, Bulgaria, Croatia, Cyprus, Czech Republic, Denmark, England, Estonia, Finland, France, Germany, Greece, Hungary, Ireland, Italy, Latvia, Lithuania, Luxembourg, Malta, Netherlands, Norway, Poland, Portugal, Romania, Russia, Slovakia, Slovenia, Spain, Sweden, Switzerland, Ukraine, and Turkey; *Middle East and Africa:* Egypt, Ethiopia, Iran, Israel, Nigeria, Saudi Arabia, South Africa, United Arab Emirates; *Americas:* Argentina, Brazil, Canada, Chile, Colombia, Mexico, Peru, Venezuela, USA; *Asia and Pacific:* Australia, Bangladesh, China, India, Indonesia, Japan, Malaysia, New Zealand, Pakistan, Philippines, South Korea, Thailand, Viet Nam
Policies	access, approval, control(s), cost(s), coverage, demand, dispensing, price(s), prescription, pricing, reimbursement, substitution
Identifiers	drug(s), medicines(s), medication(s), pharma, pharmaceutical(s)

Publications were then reviewed to identify prevailing policies, which were then categorized and reviewed by a panel of experts, comprising 25 members from around the globe. The panelists were based in academia, industry, consulting and public healthcare sectors, from countries including Australia, Brazil, China, Switzerland, Japan, Hungary, Germany, Greece, Thailand, the UK, and the USA. The experts were given a questionnaire containing the classifications and were asked to comment on and verify them. The agreed taxonomy was then used to assess the pharmaceutical care in each of the reviewed countries. In this context, countries were assessed against a checklist of policies in different domains. Very often, a country did not apply a single policy exclusively in each domain, but several of them. In such cases, all policies were recorded, but the most commonly used or the most dominant one was identified. Countries were then grouped into three income groups, using the classification of the World Bank. The underlying premise is that economic status is the most significant factor affecting pharmaceutical use and policy. The first group comprised low- and lower middle-income countries (10), the second upper middle-income countries (15) and the third high-income countries (38). Most of the countries in the third income group fall into the group of developed economies in the classification used by the United Nations, while the remaining countries in the other two income groups are classified as developing and transition economies, respectively. The overall frequency of the policies in each domain, as well as in the three income groups, was assessed to identify prevailing pharmaceutical policy patterns in relation to income and economic development status.

## Results

### Price setting policies and trends

Regarding the pricing policies applied in different countries, the review of the literature indicated that pharmaceuticals are treated differently, mainly depending on whether they are on patent, off patent or generics (or equivalent to the latter in certain settings). The main pricing policies identified include: free pricing, internal reference pricing, external (or international) reference pricing, price negotiations (accompanied often by various types of assessment of product “value”), cost plus pricing, dynamic pricing, state controls (and decisions without negotiation), conditional pricing, and price setting through compulsory reductions (applicable mainly to off-patent and generic products).

*“Free pricing”* means allowing pharmaceutical manufacturers to set product prices freely at market entry, over limited or unlimited periods. This approach to the pricing of pharmaceuticals is usually preferred by manufacturers, but risks leading to higher prices and expenditure. Hence, free pricing mechanisms are often accompanied by some form of indirect price moderation, such as rebates, and profit or price and cost controls. With *“internal reference pricing”*, prices are set based on the prices of bioequivalent or therapeutically equivalent products already on the market. Hence these marketed products directly set the benchmark price for new pharmaceuticals. The benchmark may be products that are pharmaceutically equivalent (e.g., same active pharmaceutical ingredient tested on chemical or biologic level). Based on the WHO Anatomical Therapeutic Chemical (ATC) Classification System, this benchmarking takes place between products of the same ATC5 classification. Hence, the price of an original product may be compared with generic newcomers—for instance, in the case of an original statin that becomes off patent and its generics. In other cases, it concerns comparisons at ATC4 level. Hence, it may concern more than one active substance—for instance, in the case where a new statin is compared with different original or generic statins already in the market. *“External reference pricing”* (or *international reference pricing*) means that prices of pharmaceutical products introduced to the market are benchmarked to the price of the same product in other countries. In this system, important parameters in pricing are the number and composition of reference countries and the formula for the determination of the price in the destination country. The selection of reference countries and the pricing formula indirectly determine the attained price level. The number of reference countries is usually four to eight, and they are usually in the same region as the destination country or have comparable economic and healthcare conditions. There is no general rule regarding the methods for setting prices. Some countries rely on the average of prices in all the reference countries; some may use the average of the lowest prices. Usually, to avoid distortions, ex-manufacturer prices are considered, but when these are not available retail or wholesaler prices may be used.

*“Cost plus pricing”* means prices of pharmaceutical products are calculated using a formula that may include production and/or research and development costs and a defined profit margin. Since these rules are determined individually by each country, such price assessment methods create challenges for globally active companies. *“State controls”* means the state/government and its institutions set the prices of pharmaceuticals and often make decisions without any consultation or negotiation with the manufacturers. By contrast, in many countries the prices of pharmaceutics are negotiated directly with manufacturers. It is common in such systems to determine through an assessment process the medical benefit of new relative to existing products or/and the cost-effectiveness and budget impact. In this context, some countries are using Health Technology Assessment (HTA) for pricing purposes. Recently, more systematic approaches have been proposed to associate the comparative additional benefit of a new product with its price. This approach of *“value based pricing”* has the benefit that it aims to reward manufacturers for delivering better value and is rational from the payer perspective; however, it faces many challenges in its implementation, which explain its critical reception and low adoption rate so far.

*“Conditional pricing”* means that prices are set for a period under certain conditions with a reassessment after a predefined period, during which the manufacturer needs to collect and submit new evidence on the benefits and use of the product. *“Dynamic pricing”* means prices are reduced when certain conditions are met, usually based on the time after patent expiration or on the number of alternative products available on the market. Finally, *“compulsory price reductions”* refers to compulsory cuts in the prices of original products once they lose patent protection, irrespectively of whether there is competition, or to compulsory reductions in the prices of the originals to match the prices of generic products [15]. These reductions are administratively easy, but they are arbitrary and not based on any economic or scientific evidence and rationale.

**Fig. 1:**
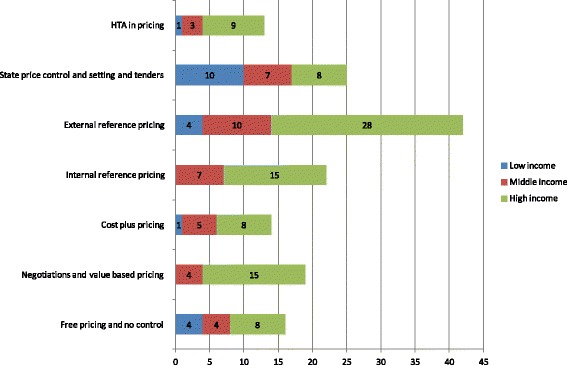
Pricing policies of on-patent pharmaceuticals

**Fig. 2:**
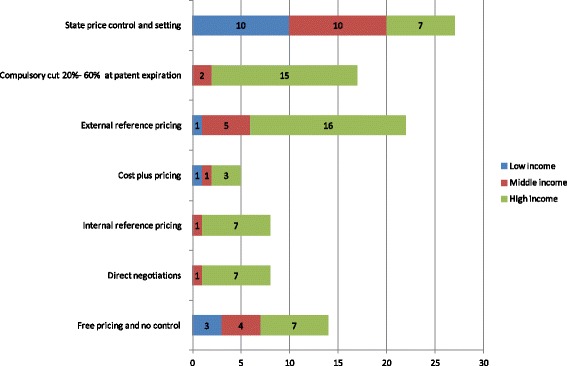
Pricing policies of off-patent pharmaceuticals

**Fig. 3:**
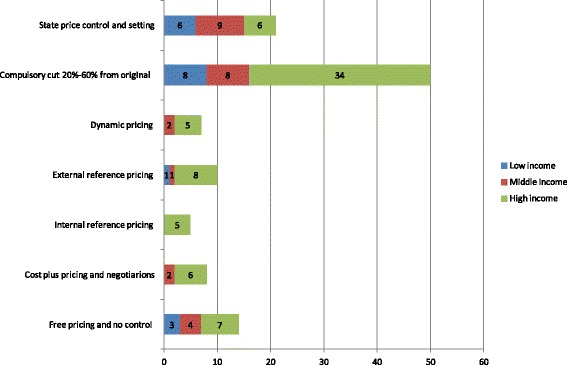
Pricing policies of generic pharmaceuticals

Additional file 2: Table S1 presents the pricing policies applied in each of the countries under consideration for the three different types of product. Figure 1 depicts the occurrence of different policies in total and by country income status. The policies sum to a total higher than the number of countries considered, as in a single country different policies may be applied for different types of products (e.g., locally produced or imported). The prevailing method for setting prices for on-patent medicines is external reference pricing, especially in high-income developed economies. This system appears simple to administer, relative to systems based on assessment of value and negotiations, and seems more objective in nature as it does not involve subjective judgments [39–42]. Therefore, is also used in many middle-income countries, alongside internal price referencing, usually at ATC4 or 3 level. Internal referencing is also used in high-income countries as a supporting mechanism. Notably, low-income countries rely more on state controls and tenders in setting prices, since they are unable to set up more sophisticated assessment mechanisms and they need to reduce prices as low as possible for reasons of accessibility and achieving the lowest possible cost. The prices for off-patent products in developed economies are driven mainly by external price referencing and compulsory price discounts, whereas in low- and middle-income countries they are determined by state controls (Fig. 2). As seen in Fig. 3, compulsory price reductions are also more common for generics in higher-income economies, whereas lower-income economies rely on state controls [12, 15, 41, 43].

### Indirect price and cost control policies and trends

Pharmaceuticals are also indirectly controlled though complementary policies aiming either indirectly at the prices or at the overall costs. The review revealed the following policies: discounts, rebates, claw-backs, pay-backs, price volume agreements, risk sharing (or managed entry) agreements, price caps or cuts and freezes, tenders for interchangeable products and company profit controls. *“Discounts”* obligate manufacturers to give regulated upfront reductions on the price depending on various conditions. *“Rebates”* are percentage refunds on individual products by the manufacturers, which may be horizontal or progressive depending on the volume of sales in a given period. *“Pay-back”* and *“claw-back”* regulations force manufacturers individually or collectively to return back to payers any amount above a predefined target at product or industry level. *“Profit controls”* limit the profits of manufacturers. *“Tenders”* refer to bidding processes applied to interchangeable product classes or groups. Tenders can only be effective if there is competition between different suppliers of interchangeable existing products. In this case, the payers can choose the most attractive offer, which is usually the cheapest. *“Price freezes”, “caps”, and “cuts”* refer to setting non-selective policies that fix or set upper limits to pharmaceutical prices for a certain period time, or introduce general relative price reductions (e.g., by a fixed percentage).

*“Price volume agreements”* are usually applied to single new products, where the price agreed is conditional on the expected number of units sold or sales made, or the market share attained. Higher usage and sales trigger lower prices, pay-backs, rebates or discounts. Hence, this approach indirectly links prices with usage and sales. They are solely financial agreements. Finally, in “*risk sharing or managed entry agreements*”*,* manufacturers assume some of the financial and effectiveness risk otherwise carried by the payers. In this context, payers and manufacturers may agree to link prices or overall expenditure for certain drugs to predefined safety, process, effectiveness or other outcomes [44]. Hence these represent a more sophisticated type of agreement based on predefined outcomes.

**Fig. 4:**
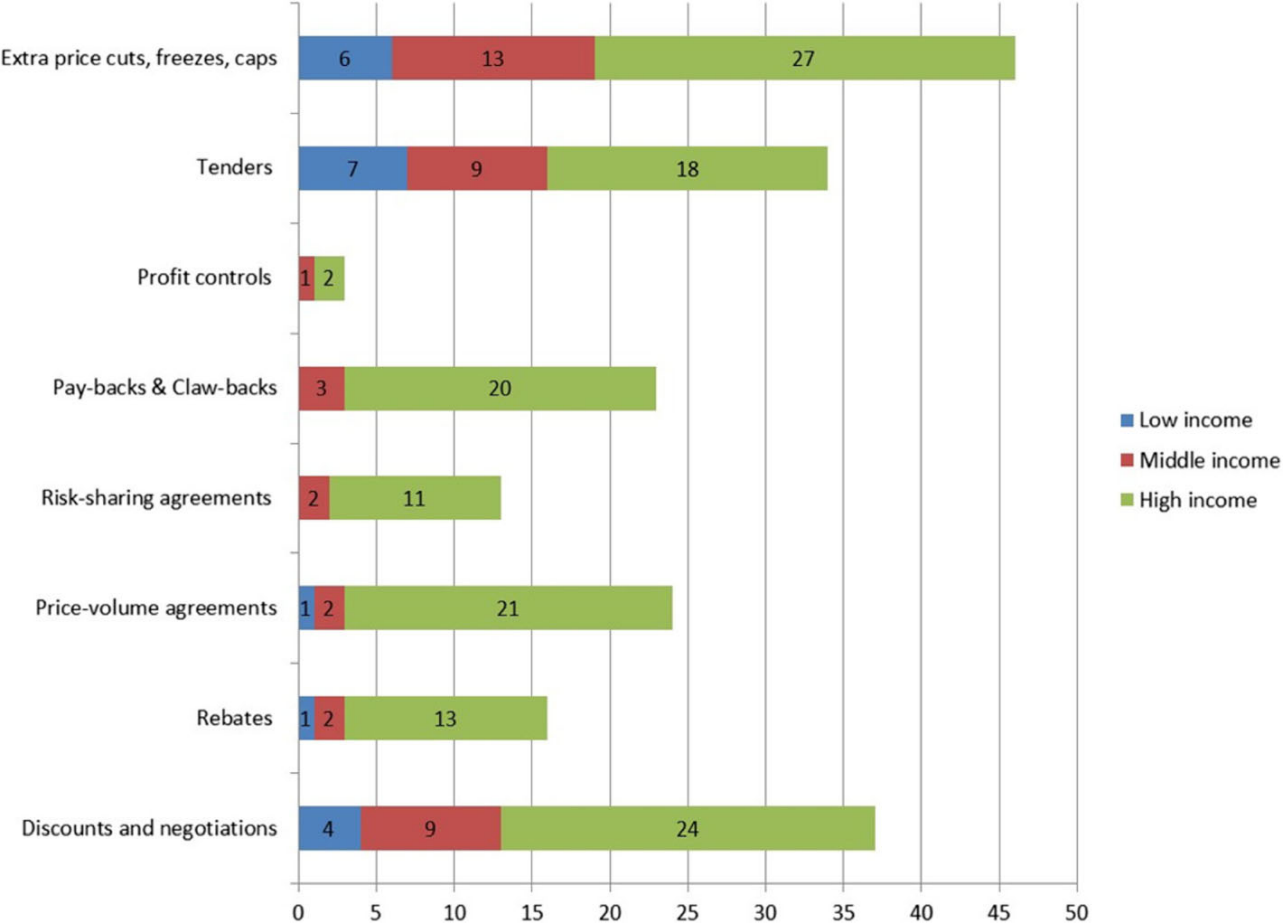
Indirect price and cost controls

Additional file 2: Table S2 in the relevant appendix presents the indirect pricing policies in the countries under consideration. As seen in Fig. 4, this review has shown that indirect-complementary price and cost controls are well-established in many developed countries [43]. Many countries exercise unscheduled price cuts, extra discounts and tenders on pharmaceuticals to control pharmaceutical expenditure. It appears that high-income countries also rely on other measures, such as price volume and risk sharing agreements, pay-backs, claw-backs and rebates. Low-income countries rely more on discounts, price cuts and tenders.

### Coverage or reimbursement policies and trends

Coverage policies determine the access of individuals to pharmaceuticals under various public schemes, which residually define what costs should be borne by patients themselves, either with out-of-pocket payments or by supplementary or complementary private insurance schemes. Three dimensions define public pharmaceutical coverage: breadth of coverage, scope of coverage, and depth of coverage[30]. *“Breadth of coverage”* defines the percentage of the population that is covered by public schemes. Many developed economies have systems providing for full breadth of coverage, where the entire population has access to pharmaceuticals through schemes funded by social security or social insurance contributions or taxation. By contrast, many emerging economies and healthcare systems have not yet achieved full breadth of coverage, and only a limited portion of the population (e.g., specific demographic, occupational or disease groups) is covered, with the remaining ones facing a high degree of self-payments. One notable case is the USA, where a significant proportion of the population have no insurance and do not qualify for Medicaid and Medicare coverage.

*“Scope of coverage”* defines the range of pharmaceutical products reimbursed by the statutory schemes. The review found that in some cases, only a very *limited number* of pharmaceutical products, such as some generics or locally produced products, are covered by public reimbursement schemes. In other cases, drugs classified in the *Essential Drug (Medicines) List (EDL)* and/or drugs used only for a range of serious or life-threatening diseases (e.g., cancer and HIV) are covered. These types of policies are common in developing economies and healthcare systems. Sometimes there are situations where on top of the EDL some *additional drugs* are covered. In systems of developed countries, a full range of products is reimbursed. In these countries, pharmaceuticals covered are often included in a *positive reimbursement list* and/or those not covered are excluded by placement on a *negative reimbursement list*. Often in many *countries health technology assessment and economic evaluation or cost-effectiveness analysis* is used to decide whether a product should be reimbursed or not.

*“Depth of coverage”* concerns the reimbursement level, that is, the proportion of the price covered by statutory schemes. *Full cost* schemes cover the total cost of the product and the patient has nothing to pay for the products being covered. *Co-payment* systems refer to situations where co-payments are covered by patients and the remaining amount by payers. These have many different forms and mechanisms. In some circumstances, they concern fixed amounts per prescription or the entire cost for inexpensive drugs below a certain level. In other cases, different rates are applied and these may also be linked to the patient’s age and socioeconomic or employment status. In other cases different rates may be applied to products for different diseases. Rates start from zero (no co-payment) for more severe and life threatening diseases and go up to 100% (full self-payment) for non-serious and lifestyle perceived conditions. Co-payment rates for chronic and other diseases fall in between these extreme values, depending on the severity of the disease and the cost of treatment.

Often, many therapeutically similar substances for the same condition exist, but have considerable variation in their retail prices. Hence, many countries have developed *“internal reference pricing systems”* to define reimbursement prices. Pharmaceuticals in this context are grouped into clusters or classes, within which they are all considered to be interchangeable. These clusters are characterized by the level of the WHO ATC Classification System adopted. In particular, clusters may be formed only at ATC5 level; thus, they may include only pharmaceutically equivalent or bio-equivalent drugs. Hence, at this level they mainly include an original product and its generics—as in the case of an original statin, which came off patent, and its generics. For instance, simvastatin with its generics may form one cluster and atorvastatin with its generics another. In other cases, they may be formed at ATC4 level with a broader definition of interchangeability. For example, a cluster may include all original statins (on and off patent) and their generics. In this case simvastatin and atorvastatin, with their generics, would form a single cluster. However, even broader (jumbo) clusters, at ATC3 level may be formed and, for example, may include b-blockers, statins and ACE inhibitors all together. In each cluster, a maximum reimbursement price is set with a low-price benchmark (e.g.,. lowest price or average of the lowest prices of the cluster). Patients are then required to pay the price differential above the reimbursement price if they prefer a specific more expensive brand.

Simple co-payment systems and internal reference systems may be applied on their own, or in certain cases may co-exist. *Protection mechanisms* are also applied in many high-income countries to protect patients from excessive co-payments. Hence, sometimes deductibles are applied, which means that a certain amount of money must be fully covered every year by the patients themselves and public reimbursement only starts at a higher utilization level. In other cases, co-payments cannot exceed a percentage of annual income. In addition, there are reduced co-payments and exemptions to protect various vulnerable groups, defined in terms of age, health status, or income (e.g., in Sweden).

Additional file 2: Table S3 summarizes current coverage policies by breadth, scope and depth of coverage in the countries studied, where there are significant differences between developed and less developed economies. In terms of breadth of coverage, high-income countries (e.g., Western Europe, Japan, South Korea, New Zealand and Australia) have almost universal coverage through public schemes. The USA is an obvious exemption to this trend, as it relies primarily on self-insurance, and Canada is also a similar case. Some countries in South America, Asia and the Middle East have in recent years improved the breadth of coverage alongside improving economic development; however, they are still lagging behind in comparison to high-income countries. Hence, self-payment and self-coverage is very prevalent, since a significant portion of the population are not covered at all by public schemes. There is another group of countries, comprising many African nations, India, Russia and Ukraine, where coverage mechanisms are severely restricted and underdeveloped. Thus, people rely mostly on self-payment or humanitarian mechanisms (Additional file 2: Table S3). Nonetheless, there are variations in this group. Brazil, for instance, has universal access to healthcare in law and there is limited coverage by public schemes [45].

**Fig. 5:**
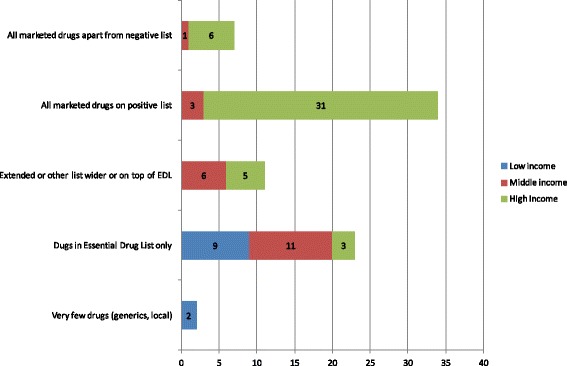
Scope of pharmaceutical coverage

**Fig. 6:**
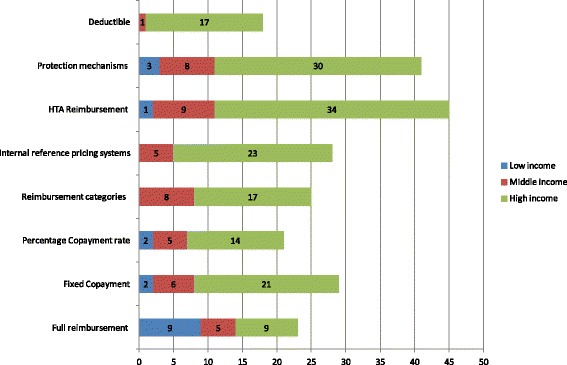
Depth of pharmaceutical coverage

In terms of the scope of coverage (Fig. 5), in high-income countries most available products in the market are covered either by being placed on a positive list, or by not being on a negative one. On the other hand, low-income and middle-income countries cover fewer pharmaceuticals, usually those on the EDL, or some locally produced. In terms of depth of coverage (Fig. 6) high-income countries attempt to control it by streamlining reimbursement and all individual cost sharing policies, while at the same time they develop mechanisms for protection and health technology assessment. Some middle-income countries imposing also impose co-payments, which are easy to implement from the administrative point of view. Low-income countries do not apply these mechanisms, because in any case coverage is very poor in terms of breadth and scope and hence such policies are unnecessary.

### Prescription and dispensing control policies and trends

Pharmaceutical expenditure is significantly driven by the quantity and mix of medications prescribed and dispensed. In recent years, many emerging policies have been focused on the control of prescribing by physicians and dispensing by pharmacists. The review identified that physician prescription behavior can be influenced by educational campaigns, prescription-aiding tools, electronic mandatory prescription systems, indicative or compulsory prescription guidelines, prescription quotas and targets, predefined prescription budgets, indicative or compulsory prescription by generic names, such as the International Non-proprietary Name (INN), prior and posterior restrictions and approvals, monitoring and benchmarking of prescribing, and finally sanctions and incentives for target attainment or guideline adherence. These policies have been classified into four main domains: education, engineering, economics and enforcement [46].

*“Educational campaigns and programs”* target physicians to inform them about efficient prescription practices. *“Prescription-aiding tools”* encourage physicians to use electronic or hard-copy catalogues to support them during the prescription to improve their knowledge and efficiency. *“Electronic mandatory prescription”* controls prescribing by mandating it to be implemented electronically and by not allowing for handwritten prescriptions. *“Indicative”* or *“compulsory prescription guidelines”* define best prescription practices, which in many cases are incorporated into electronic prescription systems. *“Prescription quotas”* and *“targets”* set prescribing limits for physicians. This could include specific rates of generics prescribed in specific drug classes or across all prescriptions. *“Predefined prescription budgets”* are budget limits for physicians that, if exceeded, will trigger a penalty or some kind of restriction and control. *“Indicative”* or *“compulsory generic prescription”* promotes or imposes prescription by generic code, such as the INN. *“Prior”* and *“posterior restrictions and approvals”* are applied to restrict the use of certain, usually expensive, drugs to a specific patient group. In this context, there is peer assessment for physicians. *“Monitoring of prescription patterns and prescription data and benchmarking”* refer to processes for analysis and benchmarking individual physicians’ prescription data and patterns, combined with feedback mechanisms. *“Sanctions and incentives”* for target and guideline adherence are supposed to improve conformance with the set standards.

In most countries, prescribing and dispensing functions are separated. Only physicians can prescribe medicines, whereas pharmacists are responsible for dispensing them and so can play a significant role in the final medicine selection at the point of dispensing. The present literature review identified many different rules used to influence drug dispensing and choice of pharmaceuticals. Sometimes, pharmacists are free to change prescriptions, even though there are no specific policies in place to promote this change (*“no other policies in place but freedom of pharmacists to change prescription”)*. In many low and lower middle-income countries in Africa, Asia, and Latin America, patients can purchase medicines directly from the pharmacist; this also applies for some developed countries (e.g., Greece) [47, 48]. In other cases, there is indicative substitution at substance level, which is often combined with soft incentives to substitute cheaper products (*“incentives in place for substitution”)*. Incentives to the pharmacists may, for instance, provide for higher dispensing compensation when the pharmacist dispenses preferred products. Other policies often provide strong incentives to promote or mandate substitution of cheaper products to be selected based on pharmaceutical, biological or therapeutic interchangeability: *“strong measures and incentives in place for substitution”*. These are used to promote the substitution and dispensing of lower-price products and include mandates, quotas, targets, claw-back and rebate mechanisms. Such systems may or may not give the physician an opportunity to prohibit substitution. With *“compulsory substitution”* the pharmacist is obliged to dispense the cheapest or one of the group of cheapest products in the store, or the cheapest in the country with a similar active substance.

**Fig. 7:**
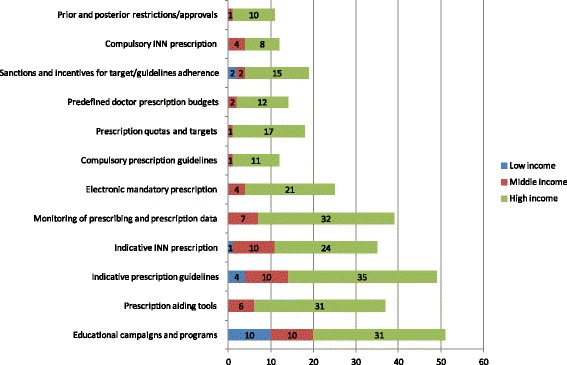
Pharmaceutical demand control policies

**Fig. 8:**
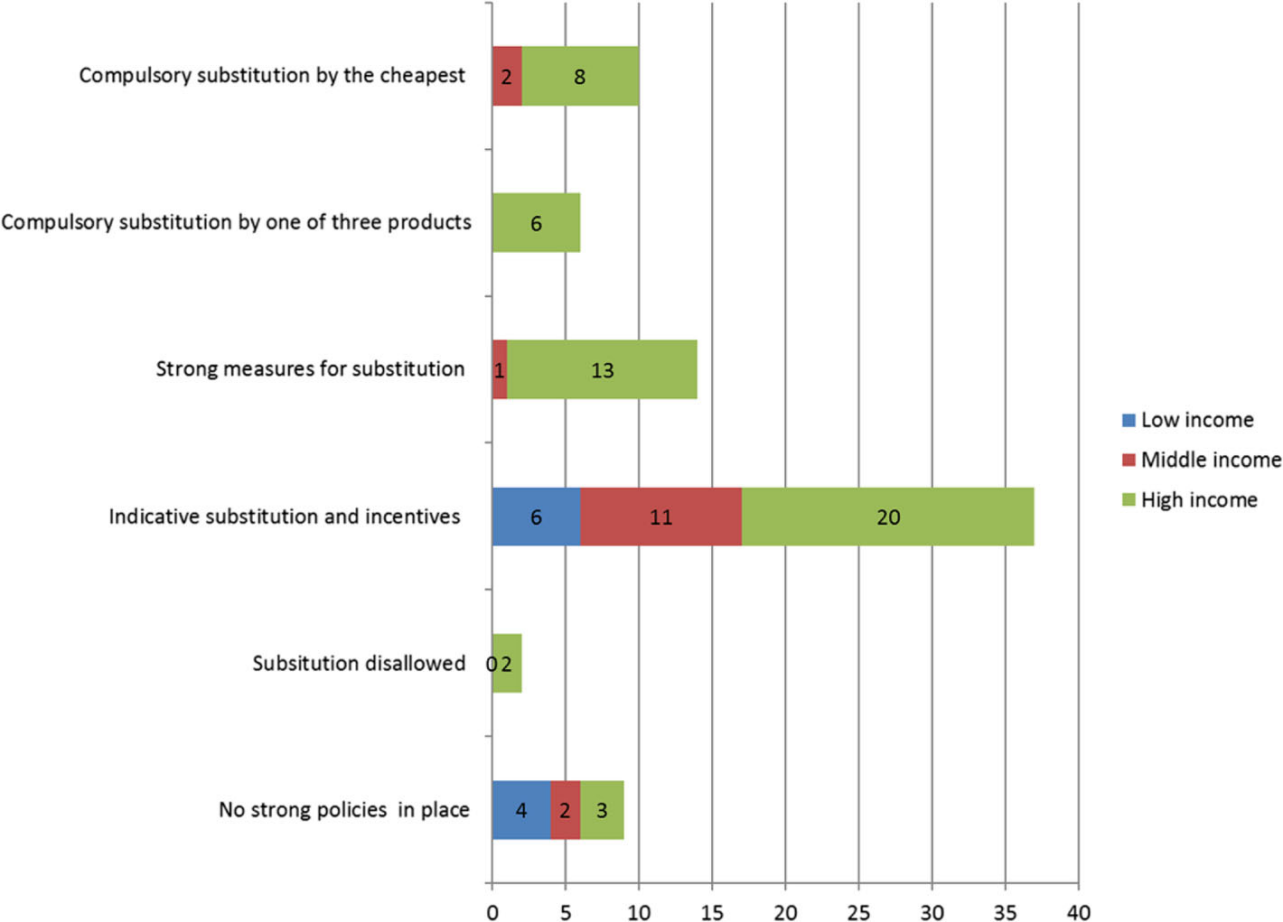
Pharmaceutical dispensing control policies

Additional file 2: Table S4 presents the policies applied for controlling demand and dispensing in the countries considered. Demand control policies are applied mainly in high-income countries where coverage and expenditure are higher (Fig. 7). Some are restrictive, others provide incentives to encourage prescribers to use evidence based management of patients, and again others incentivize cost-saving based on prescribing decisions. As shown in Fig. 8, another trend more common in developed economics is substitution for a prescribed drug and dispensing of the cheapest available product for that INN. A global trend in developed countries through demand control and dispensing policies is the promotion of generic substitution and/or generic prescribing [9, 16, 34, 49, 50]. This is carried out using a combination of guidelines, INN prescription, internal reference systems and pharmacy substitution schemes (Fig. 8).

## Discussion

In terms of pricing for on-patent drugs, the review identified that most developed countries are using external reference pricing [19, 30, 31, 34, 41, 51–58]. This appears to be a straightforward approach to apply: however, there are many caveats to be considered. Sometimes, the prices in the reference countries are not easily accessible, may not be transparent for this kind of undertaking, or may refer to differing doses, formulations and packages [16]. An additional level of complexity lies in the definition of the reference price (e.g., ex-factory, consumer price, after or before rebate, after or before VAT, impact of exchange rate, etc.) [27, 32, 36]. Given the nature of the exercise, there may be price fluctuations in the destination country even while prices in the country of origin stay constant, because of factors such as exchange rates. Hence, far-reaching interdependency and cross-referencing of countries adds further complication. External price referencing also does not account for differences between countries in income, affordability and willingness to pay [16]. Extreme pricing decisions in one country may impact the entire price system in all countries involved in the referencing chain, and may also cause parallel exports and shortages. It is a fact that the policies and priorities in one country have an impact on others.

Pharmaceutical companies have been developing strategies to deal with such challenges in pricing. For instance, companies may decide not to launch or to delay launches of products in lowest-price countries, thus strongly influencing other countries’ price levels. This approach also creates access issues. Furthermore, companies try to homogenize product prices, which is harmful for lower-income countries as they may be obliged to buy products at higher prices than those they could get under a differential pricing system. An example where high prices can be harmful are the anti-TNF alphas, where patients in most Eastern European countries have only limited access because of high prices and patient co-payment [59]. On the other hand, the introduction of external reference pricing meant that countries such as Croatia were able to launch new medicines, which they could not have done otherwise. Thus, in introducing external reference pricing systems, serious consideration is needed to define a system that will balance simplicity, transparency, access, affordability and efficiency.

Internal reference pricing, usually at ATC4 level, is common in middle- and in many high-income countries, as a supporting mechanism. This approach also faces challenges, though it seems straightforward. New products are often superior in terms of efficacy, safety, tolerance and dosing in comparison to older ones that have been on the market for some time. It is challenging to identify the proper benchmarks and then define prices relative to them, using only simple algorithms and without any kind of more complex assessment of the differences in benefit. Pricing based on health technology assessment and on value-based pricing, which is an emerging policy in some high-income economies, is linking the price to the benefit offered by new pharmaceuticals; it is thus considered a fairer approach for pricing patented pharmaceuticals [60–65]. The advantage in this case relates to the fact that a “value” is determined for the product in comparison to current standards of care, and this defines the price. The higher the perceived value the higher the price. Thus, innovation and progress beyond the current level are rewarded while “more of the same” (me-too products) should not lead to increases in price or healthcare costs. However, value-based pricing is complex in terms of the methods needed and involves assumptions that entail some degree of subjectivity, interpretation and risk taking. Current methodologies are static and do not reflect multiple product characteristics, product life-cycle developments, new data generation or other product and market changes [66]. Moreover, whether or not something is deemed an innovation really depends on the benchmark used to evaluate it, and this choice may influence any assessment. Choosing the right benchmark is not that straightforward. In the UK, where value-based pricing has been considered recently as a new pricing approach, concerns have been expressed by manufacturers regarding how it should be applied for the pricing of originators in classes where one product becomes or is already available as a generic. In addition, only a limited number of new products are seen as innovative by assessment bodies; thus, under value-based pricing it could be argued that the prices of the great majority of new medicines should be similar to those of low-cost generics or off-patent products [11, 67]. This would be a disincentive for industry to invest in such classes, which in turn could be detrimental in the long run for new innovations and for competition.

The prices for off-patent products are mainly defined by external price referencing, compulsory price reductions and state controls not based on any assessment [30, 68]. Price reduction is becoming in certain settings a commonly used approach, as it is administratively easy to implement. However, this approach lacks any guiding principle for finding the right price level. There is no economic justification or any rational argument to determine what represents a proper discount at patent expiration. Moreover, it may be argued that off-patent pharmaceutical products encapsulate a great deal of “value”, as they are established products with many years of presence and large quantities of experience and data; therefore, other approaches to their pricing could be investigated, rather than arbitrary cuts. A new approach may be needed to price patent pharmaceuticals in a way that will capture some of their residual value relative to new options.

Low- and middle-income countries rely more on state price controls, which represent the main approach for setting prices because of affordability and simplicity issues [19]. Once again, however, regulation of this kind is arbitrary and does not reflect differences in the efficacy, safety and quality of products. On the other hand, it needs to be acknowledged that these economies face challenges as they strive to increase coverage with the limited financial public resources available. Large differences between affluent and poor parts of the population can be observed in terms of access to healthcare in this setting, raising equity issues. Most of the population often has only access to locally produced products, where the quality depends on the stringency of the local regulations and control mechanisms. It is often the case that regulations are loose and quality is compromised, raising safety issues. Medicines produced by multinational companies may only be available to the more affluent or privately insured parts of the population, whereas for the rest they are unavailable or have lower penetration compared with locally produced drugs. Hence, access to innovative products is low, because of the relatively high prices and manufacturer monopolies due to intellectual property protection; as a result, there is much inefficiency and scope for improvement [19]. In many developing countries, policies are in place to promote and encourage local manufacturing (Brazil, Russia, China) and hence to increase access to innovation at a lower cost.

Low-income countries could potentially afford all medicines for their population if the prices offered were low under special arrangement. For instance, Novartis are launching a scheme in Kenya where they are making available medicines for non-communicable diseases (NCDs) at US$1/medicine/patient/month and claim that they will make a profit at this price. This approach will be of significant help in low-income countries where an appreciable percentage of the population earns US$1-2/day. Firms are making appreciable gross profits on their medicines and lowering these would appreciably help all countries achieve good access to medicines—especially as a large proportion of standard medicines are now available as generics or biosimilars. Moreover, access and quality issues could be addressed and special agreements could be implemented if drugs were made available via governmental structures, e.g., hepatitis products in Egypt.

In terms of indirect price controls, this review has shown that indirect-complementary price and cost controls are well-established in many developed countries [30, 34]. In particular, many countries apply unscheduled price cuts, extra discounts and tenders to pharmaceuticals as means of controlling pharmaceutical expenditure. It appears that high-income countries also rely on other measures, such as pay-backs, claw-backs and rebates. Such cost-containment tools are complementary to price regulation, are easy to apply, and ensure short-term savings. They also have the advantage that they reduce prices indirectly, and thus have limited spillover effects through external price reference mechanisms outside the country in which they are applied. However, if over used, it has been argued that excessive price cuts and pay-back schemes may have detrimental effects on access to medicines, they may cause product launch delays or product withdrawals and shortages, and in addition they may discourage the development or introduction of new medicines [38, 69–79]. However, it must be acknowledged that an appreciable number of new medicines are under development in Europe, despite increasing controls of this kind.

In cases where paybacks are imposed and targets of aggregated pharmaceutical expenditure have been exceeded, the entire industry is jointly liable for pay-backs. This may drive individual companies to promote and heavily advertise individual high-income products, as the pay-back payment is spread across the sector. These systems also reduce price transparency, as the actual market prices are unclear and cross-border price-comparisons become difficult. The published prices in each country may no longer reflect the actual transaction prices, as these are adjusted by the above mechanisms. Consequently, the feasibility of making valid and reliable price comparisons and constructing valid price indexes is undermined. These mechanisms may also initiate a shift towards the utilization of product-categories uncontrolled by pay-backs. This may in turn lead to an increase in overall expenditure. Furthermore, local low-margin companies often do not have the flexibility to balance the impact of price cuts or do not have the money for pay-backs, and this can lead to bankruptcies. Hence, these indirect measures need careful consideration, despite being easy to implement in practice.

In some countries, managed entry schemes, price volume and risk sharing agreements are gaining momentum [11, 44, 80–85]. These, however, face many challenges in their implementation, such as high implementation costs, issues surrounding the measurement of performance and outcomes, and the need for a suitable data and information technology infrastructure when they are outcomes-based [85]. Nonetheless, despite these drawbacks, these schemes have the advantage that they achieve savings while also enhancing access to and reimbursement for new medicines, whereas this would not happen otherwise [82].

In terms of coverage, it appears from the review that an emerging trend is the use of internal price reference systems based on ATC 4 and 5 to limit public pharmaceutical expenditure in cases where the breadth of coverage is high [16, 30, 34, 51, 53, 68, 86, 87]. This is often considered to be a component of, or a complement to generic promotion policies. Reference pricing builds on consumer price sensitivity. The maximum reimbursement price is fixed and if the patient prefers a more expensive product, they must either pay the difference (e.g., in Germany) or very rarely pay for all of it (e.g., in Spain). Hence, manufacturers should keep the price within the range of the reference price, especially for products whose demand is elastic to price, because they differ little from others and there is poor patient or physician loyalty. It has been argued that, under certain conditions, this approach may result in competition and price reductions [30].

Critics point out that, by regulating reimbursement prices, authorities might inadvertently prevent further price reductions that would be driven by the introduction of greater generic competition [30]. In addition, it has been argued that this system may also in certain cases cause detrimental effects, if it triggers too much drug switching [88–91]. On the other hand, there is evidence that this may not be the case. For instance, in the Netherlands, tendering every 3 months has led to very low prices for generics and significant generic penetration and switching, alongside savings. In Scotland, INN prescribing, market forces to achieve low prices for generics, and demand-side measures have been used to encourage generic prescription in certain product classes; this has also resulted in considerable savings and switching. In neither case has there been any compromise of the quality and safety of care [92, 93].

Cost sharing is encouraged in many high- and middle-income countries, because it is supposed to make individuals more economically responsible and thus to reduce moral hazard [30]. In economic theory, a moral hazard describes the phenomenon that people tend to take higher risks or over-consume if they do not have to face the consequences or cost of doing so. That would mean that more drugs are used than needed, because someone else other than the patients will pay for them. Cost sharing is a remedy for this issue, because individuals should become more careful consumers. However, it has been argued that such co-payments may negatively impact the welfare of lower-income individuals and may lead to underutilization [30, 94, 95].

These detrimental effects may more significant in low-income countries, where because coverage is poor, people must rely on self-coverage in order to gain access to pharmaceuticals and be protected from the possible effects of ill health. In this setting, apart from mechanisms to protect vulnerable groups and improvements in coverage, lower prices and lower co-payments appreciably enhance the use of medicines and access to care, particularly in the case of NCDs [96, 97]. In this light, the aforementioned initiatives by manufacturers to offer products at low prices may have a significant effect on access and care.

Another emerging trend in developed but also developing countries is the use of health technology assessment, or pharmacoeconomics or economic evaluation in decisions regarding the reimbursement for new products [30, 54, 62, 80, 82, 98–107]. This approach has many advantages, as it links reimbursement to the value of new products, but there are many challenges and issues to be considered. To name a few, it needs local expertise, resources, time, data and updating. There are many technical and methodological issues that remain unresolved in this field and great variations are seen in the prevailing HTA models. In addition, it necessitates a clear understanding of what the acceptability thresholds should be, and this is not always easy to define.

In terms of demand and dispensing controls, as physicians are seen as key decision makers and drivers of treatment utilization, they are in the centre of attention and many policies are focused on impacting their behavior. Pharmacists may also influence the drug taken by patients. Hence, demand control policies are becoming very common across high-income countries where coverage and expenditure are higher [15, 16, 30, 68]. These aim to promote the rational use and optimal mix of drugs, with as much generic prescribing as possible. A condition for attaining those objectives is that the quality and therapeutic equivalence of products must be assured through other pharmaceutical policies. It has often been argued that too many controls and restrictions as a result of over-regulation may lead to non-optimal and inefficient equilibriums [24, 27, 29, 30, 46]. Thus, a balanced approach and more freedom of choice and competition may achieve better control of demand and utilization in comparison to over-regulation.

## Conclusions

Pharmaceuticals provide great benefits to patients and can directly or indirectly create opportunities for significant growth and economic development. On the other hand, the associated expenditure continues to increase worldwide. Thus, governments are intervening to improve access, quality, effectiveness and efficiency of pharmaceutical use. Many different policies are used to achieve these objectives and these may be combined in different ways, but each has aspects that make its implementation more or less difficult and robust. The aim of the review was to taxonomize these policies and to assess their use. The review found that the policies used are greatly influenced by a country’s economic status. In high-income developed countries the prevailing policies include universal coverage, external reference pricing for on-patent products, discounts for generic products, indirect price and cost controls, internal price reference reimbursement systems, health technology assessment, cost-sharing mechanisms, and demand and dispensing controls. Their aim is to control the uptake of new technology, to increase generic use and to control cost.

Many middle- and low-income countries are performing poorly in terms of coverage and it is important to increase their performance in terms of pharmaceutical coverage. Essential drugs in these countries are being made available to an ever-increasing number of people, but large portions of their populations are still not covered and many products available in high-income countries are not yet provided. In this context, they rely mostly on severe price controls and tenders to control expenditure, while other policies are redundant as people carry much of the burden for pharmaceutical care through direct out-of-pocket payments. This situation indicates that, despite the economic growth in recent decades and globalization, there are still significant disparities in the access of people to medicines. Pharmaceuticals improve health and better health supports better economies. Often, they also reduce the utilization of other health care resources and hence some of the cost is offset. In this light, they can represent an investment in the health of both societies and economies.

Obviously, the present system of producing and promoting pharmaceutical use is very much oriented towards the conditions, needs and finances of high-income countries, which in most cases can afford to cover their citizens with universal schemes that provide access to most of the available products with some sort of private co-payment. Nonetheless, it appears that low- and middle-income countries lack the finances and the mechanisms to provide sufficient coverage. Economic growth means that many countries in South America and Asia in the years to come will have a middle class capable of supporting public security schemes for large portions of the population, making it easier for governments to support the remaining sectors through taxation and other financing schemes. However, this will take many decades to realize. Given their profitability margins, pharmaceutical companies could and should under agreements facilitate access and coverage by offering their products at affordable levels to these low- and middle-income countries. This approach will be good for them, as well as for the populations living in these countries and for their economics. Just as managed-entry schemes are facilitating access to medicines in high-income countries, new collaborative schemes and partnerships need to be developed for facilitating access in low- and middle-income countries. We are far away from this goal, but simply waiting for these countries to grow economically and to be able to afford medications has a significant opportunity cost and is unethical.

This study presents in a standardized manner a global overview of the policies regulating pharmaceutical use and pharmaceutical expenditure in many different countries across the globe. Its main contribution stems from the fact that, in a purely descriptive manner, it conveys four important messages. First, that pharmaceutical products are made available in a global interconnected environment where policies in one country affect many others and vice versa. Second, pharmaceutical regulation is multidimensional, with many different interrelated modalities. Third, it is a highly technical exercise and each policy domain involves a mass of details and methodological issues that are challenging to deal with. Finally, policies must reflect the priorities and economic and other conditions of the country in hand. For these reasons, it is apparent that a comprehensive and sophisticated approach is needed to the design, execution and assessment of pharmaceutical policy. There are no other papers in the literature with such a broad scope in terms of geography and policies. Obviously, the present paper is based on a descriptive and subjective selection and review of a vast literature, which is still growing and has many gaps. Much research and analysis is needed to better understand pharmaceutical markets and to develop tailor-made policies that can achieve balanced and optimal solutions in different settings.

## Additional files

Additional file 1: References by country. List of the references used to evaluate pharmaceutical policies in the countries considered. (DOCX 36 kb)

Additional file 2: Policies applied per country and policy domain. List of the tables with pharmaceutical policies used in each policy domain in the countries considered. (DOCX 62 kb)

## Abbreviations

EDL: Essential Drug (Medicines) List
INN: International Non-proprietary Name
ÖBIG: Österreichisches Bundesinstitut für Gesundheitswesen
WHO: World Health Organization
