# Supplementary material for: Comprehensive taxonomy and worldwide trends in pharmaceutical policies in relation to country income status
Source: BMC Health Serv Res. 2017 May 25;17:371. doi: 10.1186/s12913-017-2304-2 (PMC5445358; doi:10.1186/s12913-017-2304-2)
Supplement: Supplementary file 1 — References by country. List of the references used to evaluate pharmaceutical policies in the countries considered. (DOCX 36 kb) [file 12913_2017_2304_MOESM1_ESM.docx]

**Appendix A.** References by country

Multiple countries

1. OECD, Pharmaceutical Pricing Policies in a Global Market. Health Policy Studies 2008: p. 1-219.

2. Espín , J., Analysis of differences and commonalities in pricing and reimbursement systems in Europe. 2007. European Commission, Directorate General Enterprise and Industry, Directorate F, Unit F5, Office BREY 10/213, 45, Avenue D'Auderghem, B-1049 Brussels: p. 1-219.

3. Habl, C., K.A., et al., Surveying, Assessing and Analysing the Pharmaceutical Sector in the 25 EU Member States. Comissioned by European Commission, 2006. p. 1-794.

Austria

4. Arts, D., Habl, C., Leopold, C., Windisch., F., et al., Austria: Pharma Profile, N. Satterly, K. Antony, and Rosian-Schikuta I., Editors. 2007.

5. Martin, C., Pharmaceutical Country Profile Austria. 2010.

6. IMS Health., IMS Pharmaceutical Pricing & Reimbursement Concise Guide. AUSTRIA. 2011.

Belgium

7. Swaef, A, Antonissen, Y., Pharmaceutical Pricing and Reimbursement Information, C.L. Gesundheit Österreich GmbH / Geschäftsbereich ÖBIG: Sabine Vogler, Editor. 2008.

8. IMS Health, IMS Pharmaceutical Pricing & Reimbursement Concise Guide. BELGIUM. 2010.

9. Gerkens, S., Merkur, S., Belgium: Health system review. Health Syst Transit, 2010. 12(5): p. 1-266, xxv.

10. IMS Health, IMS Pharmaceutical Pricing & Reimbursement Concise Guide. BELGIUM. 2012.

Bulgaria

11. Meagher, P., Azfar, O., Rutherford, D., Governance in Bulgaria's Pharmaceutical System a Synthesis of Research Findings. 2005.

12. Andre, G., Semerdjiev, L., Pharmaceutical Pricing and Reimbursement Information: BULGARIA, Thomson T., et al., Editors. 2007.

13. Andre, G., Semerdjiev, L., PHIS Pharma Profile Bulgaria. 2010.

14. Dimova, A., et al., Bulgaria health system review. Health Syst Transit, 2012. 14(3): p. 1-186.

Cyprus

15. Golna, C., et al., Health care system in transition: Cyprus, Allin S. and Mossialos E., Editors. 2004.

16. Ashikales, X., Tsinontides, A., Pharmaceutical Pricing and Reimbursement Information: CyprusThomson T., et al., Editors. 2007.

17. Petrou, P., Pharmaceutical Health Information System PHIS Hospital Pharma Report CYPRUS Morak S., Zimmermann N., and Vogler S., Editors. 2010.

18. Theodorou, M., et al., Cyprus health system review. Health Syst Transit, 2012. 14(6): p. 1-128.

Czech Republic

19. Bryndova, L., et al., Health Care system in transition: Czech Republic, Gaskins M. and Ginneken E., Editors. 2009.

20. Zalesakova, P., PHIS Hospital Pharma 2009 :CZECH REPUBLIC, Zimmermann N., Editor. 2009.

21. Petrikova, A., et al., Description of the Rricing and Reimbursement System in the Czech Republic. in ISPOR. 2010.

22. IHS, Czech Republic Health care and pharma. 2010.

23. Vostalova, L., Experience with external reference pricing in the Czech Republic. 2011.

24. Mareckova, et al., Pharmaceuticals in the Czech Republic. 2012.

25. Bochenek, T., Reimbursement of pharmaceuticals in the Czech Republic, Slovakia and Hungary – the update on reference pricing and risk sharing. Journal of Health Policy and Outcomes Research, 2012. 2: p. 11-14.

26. Kinkorova, J., Topolcan, O., Overview of healthcare system in the Czech Republic. EPMA J, 2012. 3(1): p. 4.

Denmark

27. IMS Health, IMS Pharmaceutical Pricing & Reimbursement Concise Guide DENMARK. 2011.

28. Olejaz, M., et al., Denmark health system review. Health Syst Transit, 2012. 14(2): p. i-xxii, 1-192.

Estonia

29. Pudersell, K., et al., Pharmaceutical Pricing and Reimbursement Information ESTONIA 2007.

30. Koppel, A., et al., Estonia: Health System Review, in Health Care system in transition, Ginneken E., Editor. 2008.

31. Kanavos, P., et al., Review of the Estonian Pharmaceutical Sector: Towards the development of a National Medicines Policy, WHO, Editor. 2009.

Finland

32. Mossialos, E., Srivastava, D., Pharmaceutical Policies in Finland Challenges and Opportunities. 2008.

33. Vuorenkoski, L., Health System Review: Finland, in Health care system in transition, Mladovsky P. and Mossialos E., Editors. 2008.

34. IMS Health, IMS Pharmaceutical Pricing & Reimbursement Concise Guide: FINLAND 2011.

France

35. Ganse, E., et al., Pharmaceutical Pricing and Reimbursement Information: FRANCE, Vogler S. and Leopold C., Editors. 2008.

36. Business Monitor International., France Pharmaceuticals & Healthcare Report Q1 2010. 2009.

37. IMS Health, IMS Pharmaceutical Prilth.,cing & Reimbursement Concise Guide France 2010.

38. Chevreul, K., et al., France: Health system review. Health Syst Transit, 2010. 12(6): p. 1-291, xxi-xxii.

39. Meyer, F., Health Technology Assessment, Pricing and reimbursement in France and European collaboration. 2012. University of Tokyo.

Germany

40. Business Monitor International., Germany Pharmaceuticals & Healthcare Report Q1 2010. 2010.

41. IMS Health., IMS Pharmaceutical Pricing & Reimbursement Concise Guide GERMANY. 2011.

Greece

42. Business Monitor International., Greece Pharmaceuticals & Healthcare Report Q1 2010. 2009.

43. Economou, C., Greece: Health system review. Health Syst Transit, 2010. 12(7): p. 1-177, xv-xvi.

44. IMS Health., IMS Pharmaceutical Pricing & Reimbursement Concise Guide GREECE. 2011.

45. Natz, A., Pharmaceutical pricing: Impact of the Greek pricing decisions on other Member States and the Transparency Directive. in Shaping the Future of Healthcare in Greece: Caring, Curing, Securing2012. Athens.

Hungary

46. IMS Health., IMS Pharmaceutical Pricing & Reimbursement Concise Guide HUNGARY. 2011.

47. Gaal, P., et al., Hungary health system review. Health Syst Transit, 2011. 13(5): p. 1-266.

48. IMS Health, IMS Pharmaceutical Pricing & Reimbursement Concise Guide HUNGARY. 2012.

Ireland

49. McDaid, D., et al., Ireland:Health system review, in Health Care system in transition. 2009.

50. IMS Health, IMS Pharmaceutical Pricing & Reimbursement Concise Guide IRELAND. 2011.

51. Gorecki, P., et al., Delivery of Pharmaceuticals in Ireland Getting a Bigger Bang for the Buck, in Research Series. 2012.

Italy

52. Martini, N., Folino-Gallo, P., Montilla, S., Pharmaceutical Pricing and Reimbursement Information. ITALY Thomson T. and Satterley N., Editors. 2007.

53. Business Monitor International., Pharmaceuticals & Healthcare Report. Q1. Italy 2010. 2009.

54. Scalzo, A., et al., Italy: health care review, in Health care system in transition. 2009.

55. Marchetti, M., HTA in Italy from a national perspective to hospital based HTA. International Conference on Applied Health Economics and Mathematics. 2010. Koper.

56. IMS Health, IMS Pharmaceutical Pricing & Reimbursement Concise Guide. ITALY. 2010.

57. Toniolo, F., Mantoan, D., Maresso, A., Veneto Region, Italy. Health system review. Health Syst Transit, 2012. 14(1): p. i-xix, 1-138.

Latvia

58. Behmane, D., Viksna, A., Gulbe, A., Pharmaceutical Pricing and Reimbursement Information, LATVIA Pharma Profile, Morak S. and Vogler S., Editors. 2008.

59. Mitenbergs, U., et al., Latvia: Health system review. Health Syst. Transit, 2012. 14(8): p. xv-xxii, 1-191.

Lithuania

60. Krukiene, G., Alonderis, T., Pharmaceutical Pricing and Reimbursement Information LITHUANIA Pharma Profile, Morak S., Editor. 2008.

Luxembourg

61. European Observatory on Health Care Systems., Health care system in transition: Luxembourg. 1999.

62. Luxembourg's Ministry of Health. and Sitra, Financing Sustainable Healthcare in Europe: New Approaches for new Outcomes, in Conclusions from a collaborative investigation into contentious areas of healthcare. 2007.

63. Krippes, R., et al., Country Brief: Luxembourg, Artmann J., Editor. 2010: Brussels.

Malta

64. European Observatory on Health Care Systems, Health Care Systems in Transition: Malta. 1999.

65. Bugeja, V., The impact of EU legislation on medicines in Malta. Journal of the Malta College of Pharmacy Practice, 2008(14).

66. Kiriasis, S., Pricing of medicinal products and reimbursement systems in EU. in GPIE. 2008. Malta.

67. Pulis, I., Pharmaceutical Health Information System PHIS Hospital Pharma Report. 2009 MALTA, Morak S., Vogler S., and Zimmermann N., Editors. 2009.

Netherlands

68. Zuidberg, C., The pharmaceutical system of the Netherlands. 2010: Viena.

69. Schafer, W., et al., The Netherlands: health system review. Health Syst Transit, 2010. 12(1): p. v-xxvii, 1-228.

70. IMS Health, IMS Pharmaceutical Pricing & Reimbursement Concise Guide NETHERLANDS. 2011.

Poland

71. Leopold, C., Poland’s Reimbursement System: Strengths & Weaknesses. in HAI Workshop. 2009. Warsaw.

72. IMS Health, IMS Pharmaceutical Pricing & Reimbursement Concise Guide POLAND. 2010.

73. Sagan, A., et al., Poland health system review. Health Syst Transit, 2011. 13(8): p. 1-193.

74. IMS Health, IMS Pharmaceutical Pricing & Reimbursement Concise Guide Poland. 2012.

Portugal

75. IMS Health, IMS Pharmaceutical Pricing & Reimbursement Concise Guide PORTUGAL. 2011.

76. Barros, P.P., Machado, S.R., Simoes, J.A., Portugal. Health system review. Health Syst Transit, 2011. 13(4): p. 1-156.

Romania

77. Vla˘descu, C., Scîntee, G., Olsavszky, V., Romania: Health systemreview, Allin S. and Mladovsky, Editors. 2008.

78. Leopold, C.,Vogler, S., Access to essential Medicines in Romania. 2010, ÖBIG Forschungs und Planungsgesellschaft.

Slovakia

79. Mazag, J., Pharmaceutical Pricing and Reimbursement Information SLOVAKIA Pharma Profile. 2007.

80. Kaló, Z., Docteur, E., Moïse, P., Pharmaceutical Pricing and Reimbursement Policies in Slovakia. 2008.

81. Szalay, T., et al., Slovakia health system review. Health Syst Transit, 2011. 13(2): p. v-xxiii, 1-174.

Slovenia

82. Hindle., D., A comparison of health insurance in Slovenia and Croatia. Australian Health Review. 2003. 26(1).

83. Albreht, T., et al., Slovenia Health system review. 2009.

Spain

84. Voglera, S., Espinb, J., Habla C., Pharmaceutical Pricing and Reimbursement Information (PPRI) – New PPRI analysis including Spain. Pharmaceuticals Policy and Law 2009. 11: p. 213–234.

85. Business Monitor International., Spain Pharmaceuticals & Healthcare Report Q4 2009. 2009.

86. Garcia-Armesto, S., et al., Spain: Health system review. Health Syst Transit, 2010. 12(4): p. 1-295, xix-xx.

87. Ferre, P., Pharmaceutical Pricing & Peimbursement system in spain. in PPRI. 2011. Viena.

88. IMS Health, IMS Pharmaceutical Pricing & Reimbursement Concise Guide SPAIN. 2011.

Sweden

89. Lakemedelsformansnamnden (LFN). The Swedish Pharmaceutical Reimbursement System. 2007.

90. IMS Health, IMS Pharmaceutical Pricing & Reimbursement Concise Guide SWEDEN. 2010.

91. Anell, A., Glenngard A.H, Merkur, S., Sweden health system review. Health Syst Transit, 2012. 14(5): p. 1-159.

United Kingdom

92. Simon, L.I., Health Care Financing Policies of Canada, The United Kingdom and Taiwan. 2006.

93. Business Monitor International, United Kingdom Pharmaceuticals & Healthcare Report Q1 2010. 2009.

94. IMS Health, IMS Pharmaceutical Pricing & Reimbursement Concise Guide UNITED KINGDOM. 2010.

95. Boyle, S., United Kingdom (England): Health system review. Health Syst Transit, 2011. 13(1): p. 1-483, xix-xx.

96. Goldsmith, R., Health Care System Structure and Delivery in the Republic of Korea Considerations for Health Care Reform Implementation in the United States. 2012.

Norway

97. Johnsen, J., Norway:health care Review, in Health care systems in transition. 2006.

98. IMS Health., IMS Pharmaceutical Pricing & Reimbursement Concise Guide Norway 2012.

Switzerland

99. European Observatory on Health Care Systems, Health Care Systems in Transition:Switzerland. 2000.

100. IMS Health, IMS Pharmaceutical Pricing & Reimbursement Concise Guide SWITZERLAND. 2010.

Croatia

101. Mastilica, M., Stampar, A., Health Care System in Croatia

102. Turek, S., Reform of Health Insurance in Croatia.,, Government of the Republic of Croatia., Editor. 1999, Zagreb,: Croatia,.

103. Harvey, K., Kalanj, K., Stevanoviæ, R., Croatian Pharmaceutical Sector Reform Project: Rational Drug Use. Croatian Medical Journal,, 2004. 45,(5,): p. 611-619,.

104. Mastilica, M., KuBe, S., Croatian healthcare system in transition, from the perspective of users. British Medical Journal. 2005. 331 p. 223–7.

105. Voncina, L, et al., Croatia: Health system review Health Systems in Transition. 2006. p. 1–108.

106. Lamza – Maronić, M., Glavaš, J., Pharmaceutical market in the Republic of Croatia. Med glas 2008. 5(1): p. 44-48.

107. Mihaljek., M., Health Care Policy and Reform in Croatia: How to see the forest for the trees. 2009.

108. Voncina, L., Strizrep, T., Croatia: 2009/2010 pharmaceutical pricing and reimbursement reform. Eurohealth., 2010. 16(4).

109. Golem, A.Z., Croatia Pharmaceutical Country Profile.,. 2011.

110. Vogler, S., et al., Comparing pharmaceutical pricing and reimbursement policies in Croatia to the European Union Member States. Croat Medical Journal. , 2011. 52 p. 183-97.

111. Huić, M., The current status of HTA in Croatia. 2011. Zagreb, Croatia.

112. Ostoji, R., Bilas, V., Franc, S., Challenges For Health Care Development in Croatia. Coll. Antropol. 2012. 36 (3): p. 707–716.

113. Kovač., N., Is Croatian Healthcare System ready to perform in the market conditions prevailing in the European Union? . Jel, 2012. 115(118).

114. Culig, J., Risk Sharing Policy in Pharmaceutical Pricing Implemented in Croatia. in ISPOR 16th: The use of risk sharing in pharmaceutical pricing and reimbursement decision in central & eastern Europe. 2013. Dublin.

Russia

115. Veldanova, M., The Russian pharmaceutical market. in Ega Annual Conference 2007. Instabul.

116. Toumi, M., Drugs Pricing in Russia. 2009. Prague.

117. Odabashian, A., Recent developments in Russian pricing regulation: new challenges for international pharma manufacturers. 2009.

118. Shulyak, S., Russian Pharmaceutical Market: Results of 2010. 2010.

119. Gilmore-Halliwell, B., Emerging Markets Due Diligence: Latin America, Middle East, Russia & Africa. in SLA-2010. 2010. New Orleans.

120. IMS Health, IMS Pharmaceutical Pricing & Reimbursement Concise Guide RUSSIA. 2011.

121. Railean, L., Country Report Russia. Pharmaceurical Market Europe, 2011.

122. Popovich, L., et al., Russian Federation. Health system review. Health Syst Transit, 2011. 13(7): p. 1-190, xiii-xiv.

123. Cegedim, Russia 2012: The Pharmaceutical Market & New Legislation - Building for Growth in a Fast Changing Marketplace. 2012.

Ukraine

124. Betliy, O., Kuziakiv, O., Onishchenko, K., The evaluation of health care system in Ukraine in the context of structural and quality-enhancing reforms. 2007: Moscow.

125. Arzinger, Pharmaceutics and Health Care: regulatory framework in Ukraine. 2010.

126. Andriciuc, C., Country Report – Ukraine, IDF, Editor. 2010.

127. Kitsenko, G., et al., Drug Law And Practice: Application to Harm Reduction In Ukraine, in PRAR. 2010.

128. Lekhan, V., Rudiy, V., Richardson, E., Ukraine: Health system review. Health Syst Transit, 2010. 12(8): p. 1-183, xiii-xiv.

129. Petrov, Y., Buchko, T., Ukraine: Recent Developments in Legal Regulation of Medicine and Healthcare. 2012.

130. Zalis’ka, O., Mandrik, O., Using HTA Elements on Inpatients in Ukraine. in Manual, Textbooks, Educational Program on Pharmacoeconomics. 2012. Ukraine.

131. Kanavos, P., Üstel, S., Costa-Font, J., Pharmaceutical Reimbursement Policy in Turkey, in Healthcare/Pharmaceutical Spending and Pharmaceutical Reimbursement Policy in Turkey. 2005: London.

Turkey

132. Keskinaslan, A., Assessment, pricing, and reimbursement of new health technologies: Innovative reimbursement models: Examples from health insurance systems in Asia. in International Conference for Improving Use of Medicines – ICIUM 2011. 2011. Antalya,Turkey.

133. IMS Health, IMS Pharmaceutical Pricing & Reimbursement Concise Guide TURKEY. 2011.

134. Tatar, M., et al., Turkey. Health system review. Health Syst Transit, 2011. 13(6): p. 1-186, xiii-xiv.

135. IMS Health, Country Report Turkey. Pharmaceutical Market Europe, 2011.

136. IMS Health, IMS Pharmaceutical Pricing & Reimbursement Concise Guide Turkey. 2012.

Egypt

137. Materia, E., Riva, G., Egyptian Health System.

138. Elmahdawy, M., Pros & Cons of pricing and peimbursement system in Egypt. in ISPOR.

139. Bayoumi, A., Pharmaceutical Pricing Policy in Egypt. 2008. Soudi Arabia.

140. UBIC Consulting, The Egyptian Pharmaceutical Industry. 2010.

141. Hosseiny, N., The Egyptian Health care System Past and Future. 2010.

142. Diaa, M., Egypt: Pharmaceutical Country Profile. 2011.

143. Elsalam, A., Measuring Transparency in the Egyptian Pharmaceutical System. 2011.

144. Shady, A., Egyptian Health Report. 2012.

Ethiopia

145. Ababa, A., Drug Financing in Ethiopia. 2007.

146. WHO, Ethiopia: Country Pharmaceutical Profile. 2009.

147. El-Saharty S., et al., Ethiopia: Improving Health Service Delivery. 2009.

148. Ababa, A., Ethiopia's Fourth National Health Accounts 2007-2008, M.O.H. Federal Democratic Republic of Ethiopia, Editor. 2010.

149. Wamai, R., Reforming health systems: the role of NGOs in Decentralization – lessons from Kenya and Ethiopia. 2010, Harvard School of Public Health: Boston.

150. Federal Democratic Republic of Ethiopia and Ministry of Health., Health Sector Development Programme IV 2010/11 – 2014/15, Ministry of Health., Editor. 2011.

151. Surafel, F., Assessing and Improving Ethiopia’s Health Care Services. 2012, Barrett Honors College., Arizona State University.

152. Erhun, W.O., Babalola, O.O., Drug Regulation and Control in Nigeria: The Challenge of Counterfeit Drugs. Journal of Health & Population in Developing Countries;, 2001. 4(2): p. 23-34.

Nigeria

153. Auton, M., Adegoke, C., Medicines Prices In Nigeria. 2006.

154. Soyibo, A., Olaniyan, O., Lawanson, A., National Health Accounts of Nigeria 2003- 2009.

155. UNIDO, Pharmaceutical Sector Profile: Nigeria. 2011: Viena.

South Africa

156. Coovadia, H., et al., The health and health system of South Africa: historical roots of current public health challenges. Lancet, 2005.

157. Schaay, N., Sanders, D., South African Health Review 2008., in nternational Perspective on Primary Health Care Over the Past 30 Years. . 2008.

158. Harrison, D., An Overview of Health and Health care in South Africa 1994 – 2010: Priorities, Progress and Prospects for New Gains. 2009.

159. Gray, A.L., Medicine Pricing Interventions – the South African experience. Southern Med Review, 2009. 2(2): p. 15-19.

160. School of Public Health & Family Medicine, Who pays for health care in South Africa? 2009, Health Economics Unit, School of Public Health & Family Medicine University of Cape Town

161. Lawn, J., Kinney, M., Health in South Africa. The Lancet, 2009.

162. Coovadia, H., et al., The health and health system of South Africa: historical roots of current public health challenges. Series, 2009.

163. World Bank. Pharmaceutical Sector Governance in the Middle East and North Africa Region – A Regional Review by the World Bank. in Workshop on Governance of Pharmaceuticals in MENA. 2010. Amman, Jordan.

164. Zhao, F., Opportunities and Challenges: Africa Pharmaceutical Sector. 2010.

165. Chester, E., South African Healthcare System. 2010.

166. Babad, Y., Horev, T., The Israeli Healthcare System: from Health Funds Dominance to a National Health Insurance Law. in International Health Seminar – ICA. 2002.

Israel

167. Rosen, B., Merkur, S, Israel: Health system review Health Systems in Transition. 2009 p. 1–226.

168. Chernichovsky, D., Israel's Healthcare System.,. 2011.

169. OECD, OECD reviews of health care quality:Isreal. 2012.

United Arab Emirates

170. WHO, Health System Prfile Inited Arab Emirates. 2006.

171. Kasteng, F., Wilking, N., Jönsson, B., Patient Access to Cancer Drugs in Nine Countries in the Middle East, 3P Relation Consulting, Editor. 2008: Dubai, United Arab Emirate.

172. Ahdab, A. O., A Glimpse of Pricing and Reimbursement in the UAE. in ISPOR Arabic Network Forum. 2012.

173. WHO, Health System Profile: Soudi Arabia. 2006.

Saudi Arabia

174. Al-Sultan, M., How to Integrate Pharmacoeconomiic Datta iin tthe Miiddlle Eastt Counttriies: The Status in Saudi Arabia. 2008.

175. Issa, N., I. Al-Ammar, M., Mostafa, S., Healthcare and Pharmaceutical Industries in Saudi Arabia. 2009.

176. Albejaidi, F., Healthcare System in Saudi Arabia: An Analysis of Structure, Total Quality Management and Future Challenges. Journal of Alternative Perspectives in the Social Sciences 2010. 2: p. 794-818.

177. Business Monitor International, Saudi Arabia A pharamceuticals & health care report Q3 2010. 2010.

178. Saggabi, A.A., Pros & Cons of Pricing & Reimbursement: Saudi Arabia Health Care System in ISPOR. 2011. Saudi Arabia.

179. Almalki, M., Fitzgerald, G., Clark, M., Health care system in Saudi Arabia: an overview. EMHJ, 2011. 17(10).

Iran

180. Basmenji, K., Pharmaceuticals in Iran: an Overview. Arch Iranian Med 2004. 7(2): p. 158-164.

181. Couper, I.D., Medicine in Iran: A brief overview, in Open Forum 2004. p. 5.

182. Langenbrunner, J., et al, Islamic Republic of Iran Health Sector Review. 2007.

183. Mehrdad, R., Health System in Iran. JMAJ 2009. 52(1): p. 69-73.

184. Larijani, B., et al., Health Policy Making System in Islamic Republic of Iran: Review an Experience. Iranian J Publ Health, , 2009. 38(1): p. 1-3.

185. Business Monitor International, Iran Pharmaceuticals & Healthcare Report Q2 2009. 2009.

186. Davari, M., Haycox, A., Walley, T., The Iranian Health Insurance System; Past Experiences, Present Challenges And Future Strategies. Iranian J Publ Health, 2012. 41(9): p. 1-9.

187. Cheraghali, A., Biosimilars; a unique opportunity for Iran national health sector and national pharmaceutical industry. Journal of Pharmaceutical Sciences 2012. 20(35).

188. Davari, M., Haycox, A., Walley, T., Health Care Financing In Iran; Is Privatization A Good Solution? Iranian J Publ Health, , 2012. 41(7): p. 14-23.

Argentina

189. IMS Health, IMS Pharmaceutical Pricing & Reimbursement Concise Guide ARGENTINA. 2011.

190. Augustovski, F., et al., Implementing pharmacoeconomic guidelines in Latin America: lessons learned. Value Health, 2011. 14(5 Suppl 1): p. S3-7.

Brazil

191. Business Monitor International, Brazil Pharmaceuticals & Healthcare Report Q1 2010. 2009.

192. Bradesco, Brazil Pharma. 2011.

193. IMS Health, IMS Pharmaceutical Pricing & Reimbursement Concise Guide Brazil. 2011.

194. Ferraz, M.B., Soarez, P.C., Zucchi, P., Health technology assessment in Brazil: what do healthcare system players think about it? Sao Paulo Med J, 2011. 129(4): p. 198-205.

195. Brazil Works, Brazil’s Healthcare System: Towards Reform? 2012.

196. IMS Health, IMS Pharmaceutical Pricing & Reimbursement Concise Guide Brazil 2012.

Chile

197. Hartwig, G., Quirland, A., Chilean Pharmaceutical & Medical Devices Market Overview 2009.. p. 128.

198. Pinto, P., Chile: Pharmaceutical Industry Overview. 2009.

199. Missoni, E., Solimani, G., Towards Universal Health Coverage: the Chilean experience. 2010.

200. IMS Health, IMS Pharmaceutical Pricing & Reimbursement Concise Guide CHILE. 2011.

Colombia

201. Giedion, U., Uribe, M.V., Colombia’s Universal Health Insurance System :The results of providing health insurance for all in a middle-income country. Health Affairs, 2009. 28(3).

202. Ibarra, A., Asociados, S.A., Colombian Market Report for Medical Devices and Pharmaceutical Products. 2010. p. 129.

203. Alvarez, L., Colombian Health System Reform. 2010, Universidad de Antioquia. . Medellin, Colombia.

204. Benavide, G., Policy Analysis: “Feasibility of Health Technology Assessment as a tool in making decisions on health in Colombia”. 2011, University of London: London.

205. Vacca, C., Acosta, A., Rodriguez, I., International reference prices and cost minimization analysis for the regulation of medicine prices in Colombia. Value Health, 2011. 14(5 Suppl 1): p. S16-9.

206. Vargas-Zea, N., et al., Colombian Health System on its Way to Improve Allocation Efficiency—Transition from a Health Sector Reform to the Settlement of an HTA Agency. Value in Health Regional Issues, 2012(1): p. 2 1 8 – 2 2 2.

207. Pinzon, A., Health in Colombia: a system in crisis. Canadian Medical Association or its licensors, 2012. 184(6).

208. Yepes Lujan, F., Colombia: A Healthcare System in Crisis, in Essay Series: Healthcare Systems – Colombia. 2012.

Mexico, Venezuela

209. OECD, OECD Reviews of Health Systems: Mexico. 2005.

210. Moïse, P., Docteur, E, Pharmaceutical Pricing and Reimbursement Policies in Mexico. 2007.

211. Moïse, P., Docteur, E., Pharmaceutical Pricing and Reimbursement Policies in Mexico. 2007.

212. Business Monitor International, Mexico Pharmaceuticals & Healthcare Report Q1 2008. 2007.

213. Laurell, A., Health System Reforming in Mexico: A critical Review. International Journal of Health Services, 2007. 37(3): p. 515-535.

214. Business Monitor International, Mexico Pharmaceuticals & Healthcare Report Q1 2010. 2009.

215. IMS Health, IMS Pharmaceutical Pricing & Reimbursement Concise Guide Mexico 2012.

Peru

216. Cornejo, EM., Medicine Prices, Availability, Affordability and Price Components in Peru. 2007.

217. Madden, J.M., et al., WHO/HAI Medicine Prices and Availability survey validation: Measuring medicine prices in Peru: validation of key aspects of WHO/HAI survey methodology. Rev Panam Salud Publica 2010. 27(4).

218. Zegarra, V., Republic of Peru, Pharmaceutical Country Profile., 2012.

219. Francke, P., Peru’s Comprehensive Health Insurance and New Challenges for Universal Coverage, U.H.C.S. Series, Editor. 2013, The World Bank: Washington DC.

220. Seinfeld, J., Montañez, V., Besich, N., The Health Insurance System in Peru: Towards a Universal Health Insurance, 2013.

Canada

221. Marchildon, G., Canada: Health System Review. 2005. 7(3).

222. Business Monitor International, Canada Pharmaceuticals & Healthcare Report Q1 2010. 2009.

223. IMS Health, IMS Pharmaceutical Pricing & Reimbursement Concise Guide CANADA. 2011.

USA

224. IMS Health, IMS Pharmaceutical Pricing & Reimbursement Concise Guide USA. 2011.

Australia

225. Hindle, D., Acuin, L., Valera, M., Health Insurance in the Philippines: bold policies and Socio-economic realities. Australian Health Review, 2001. 2(2).

226. Healy, J., Sharman, E., Lokuge, B., Australia: Health system review. 2006. 8(5): p. 1-158.

227. Business Monitor International, Australia Pharmaceuticals & Healthcare Report Q1 2010. 2009.

228. IMS Health., IMS Pharmaceutical Pricing & Reimbursement Concise Guide AUSTRALIA. 2011.

New Zealand

229. Clark, T., PharmaHandbook: A Guide to the International Pharmaceutical Industry, Taylor J., Editor. 2006: New Orleans.

China

230. World Bank, A Generic Drug Policy as Cornerstone to Essential Medicines in China, in China Health Policy Notes. 2010.

231. IMS Health, IMS Pharmaceutical Pricing & Reimbursement Concise Guide, China. 2010.

232. KPMG, China's pharmaceutical industry - Poised for the giant leap. 2011.

233. Ngorsuraches, S., et al., Drug reimbursement decision-making in Thailand, China, and South Korea. Value Health, 2012. 15(1 Suppl): p. S120-5.

234. Chang, N.W., A Comparison of Health Care Reform in Taiwan, China, and United States, G.S. University, Editor. 2013.

India

235. Kumra, G., Mitra, P., Pasricha, C., India Pharma 2015 Unlocking the Potentila of the Indian Pharmaceuticals Markets, in Pharmaceuticals & Medical Products Practice, M. Company, Editor. 2007.

236. Thatte, U., Hussain S., Evidence-Based Decision on Medical Technologies in Asia Pacific: Experiences from India, Malaysia, Philippines, and Pakistan. Value in Health, 2009. 12(3).

237. PricewaterhouseCoopers., Global pharma looks to India: Prospects for growth, in Pharmaceuticals and Life Sciences, PricewaterhouseCoopers, Editor. 2010.

238. IMS Health, IMS Pharmaceutical Pricing & Reimbursement Concise Guide INDIA. 2011.

239. Balasubramaniam, P., Bartlett, H., Seth, V.Y.a.K., Universal Health Care Systems Worldwide:16 International Case Studies in High Level Expert Group Report on Universal Health Coverage for India 2011.

240. Kumbhar, R., India, Sector Review Pharma. 2012.

241. IMS Health, IMS Pharmaceutical Pricing & Reimbursement Concise Guide India. 2012.

Indonesia

242. Plianbangchang, S., Country Cooperation Strategy Indonesia, WHO, Editor. 2008.

243. Utomo, T., The Pharmaceutical Patent Protection Impact on Indonesia Drugs Price. Mimbar Hukum, 2009. 21(3): p. 409-628.

244. Wang, et al., Private Sector Health in Indonesia: A Desk Review. 2009: Bethesda,.

245. Trisnantoro, L., Indonesian Health Reform in a decentralized system. 2009.

246. World Bank, Indonesia Health Sector Review, in Pharmaceuticals: Why reform is needed. 2009.

247. Business Monitor International, Indonesia Pharmaceuticals & Healthcare Report Q3 2010. 2010.

248. Thabrany, H., Indonesian Health Care System, Policy and The Road to Universal Health Coverage. in ISPOR. 2012. Berlin.

Bangladesh

249. Vaughan, J., Karim, E., Buse, K., An overview of the Health Care System in Bangladesh. Journal of Public Health Medicine, 2000. 22(1): p. 5-9.

250. Anwar, I., Bangladesh Health System in Transition: Selected Articles, 11, Editor. 2009.

251. Limited, D.I., Bangladesh National Health Accounts, 1997-2007, R.P. Rannan-Eliya, Editor. 2010.

252. Chowdhury, O. H., Osmani, S.R., Towards Achieving the Right to Health: The Case of Bangladesh. The Bangladesh Development Studies, 2010. XXXIII(1 & 2).

253. Anamul, H., Zahedul, A., Business Analysis of Pharmaceutical Firms in Bangladesh: Problems and Prospects. Journal of Business and Technology (Dhaka), 2011. VI(01).

254. Mahmood, S., Health Systems in Bangladesh. Health System and Policy Research, 2012. 1(1:1).

255. Khandakar Safwan, S., An Overview of the Pharmaceutical Sector in Bangladesh. Stock Brokerage LTD, 2012.

Pakistan

256. Ghaffar, A., Kazi, B., Salman, M., Health care systems in Transition III. Pakistan, Part I. An overview of the health care system in Pakistan. Journal Of Public Health Medicine, 2000. 22(1): p. 38-42.

257. Nishtar, S., Health Systems in Pakistan – a Way Forward Pakistan. in Pakistan’s Health Policy Forum and Heartfile. 2006. Islamabad.

258. Regional Health Systems Observatory- EMRO., Health System Profile Pakistan. 2007.

259. Jooma, R., Pakistan Pharmaceutical Country Profile 2010.

260. Business Monitor International, PAKISTAN PHARMACEUTICALS & HEALTHCARE REPORT Q3 2010. 2010.

Philippines

261. Juban, N., et al., Pharmaceutical Sector Scan Framework Overview of Key Pharmaceutical Sector Data Philippines. 2010.

262. Business Monitor International, Philippines Pharmaceuticals & Healthcare Report, B.M. International, Editor. 2010.

263. Country Health Information Profile: Philippines. 2011.

264. Guerrero, M., Philippines Pharmaceutical Industry, Industry Studies Department Board of Investments., Editor. 2011.

265. Romualdez, J.A., et al., The Plipippines Health System Review, Asia Pacific Observatory on Health Systems and Policies., Editor. 2011.

266. WHO, Philippines Pharmaceutical country Profile. 2011.

267. IMS Health and Pharmaceutical & Health Care Association of the Philippines., Philippines Pharmaceutical Industry. 2012.

268. WHO and Ministry of Health, Philippines health service delivery profile. 2012.

Thailand

269. Sakunphanit, T., Universal Health Care Coverage Through Pluralistic Approaches: Experience from Thailand, Social securIty extension initiatives in east ASIA 2006.

270. Antos, J., Taylor, W., Health Care Financing in Thailand: Modeling and Sustainability. 2007: Bangkok,.

271. Kelly, E., Thailand’s Pharmaceutical Industry: An Update, in The American Chamber of Commerce of Thailand. 2007, Thai - American Business.

272. Tangcharoensathien, V., et al., Achieving universal coverage in Thailand:What lessons do we learn? 2007, Health System Knowledge Network.

273. Wibulpolprasert, S., Mobilization of Domestic Resources for Essential Drugs in Developing Countries: Case Study from Thailand. 2008, Ministry of Public Health, .

274. Sooksriwong, C., et al., Medicine prices in Thailand: A result of no medicine pricing policy. Southern Med Review 2009. 2(2).

275. Thammatach-aree, J., Health systems, public health programs, and social determinants of health Thailand, in World Conference on Social Determinants of Health. 2011: Rio De Janeiro.

276. WHO , Country Cooperation Strategy at a glance: Thailand. 2012.

277. WHO, Thailand at a glance, in Country Cooperation Strategy. 2012.

Malaysia

278. Yadav, H., Ghani, S.N., The changing role of primary health care in Malaysia - The past and the future. JUMMEC, 2001. 1.

279. Merican, M.I, Rohaizat, Y., Haniza, S., Developing the Malaysian Health System to Meet the Challenges of the Future. Med J Malaysia, 2004. 59(1).

280. Badar, Z.U.D., et al., A survey of medicines pricing availability, affordability, and price components in Malaysia using the WHO/HAI methodology W.H.O.W.A.H.A.I. (HAI), Editor. 2005: Malaysia.

281. Health, M.o., Country Report of Malaysia. 2006: Malaysia.

282. Quek, D.K.L., Health Care Costs & Challenges for Malaysia. 2006 (Berita March Issue).

283. Ren, H.D., Malaysian Healthcare: Where are We Heading? 2006.

28*4.* Y u, C.P., Whynes, D.K.,Sach, T.H, "Equity in health care financing: The case of Malaysia." International journal for equity in health 7.1 (2008): 15.285.

285. Hamidy, M.B.A., The Malaysian Healthcare system. 2008.

286. Quek, D.K.L., The Malaysian Health Care System: A Review. 2008.

287. Whitepaper, F.S., Overview: Malaysian Healthcare Biotechnology, T.M.H.B. Sector, Editor. 2009.

288. BMI, Malaysia Pharmaceuticals Healthcare Report Q3 2010, in Part of BMI’s Industry Report & Forecasts Series. 2010.

289. OECD, OECD Review of Innovation in Southeast Asia: Country Profile of Innovation: Malaysia. 2011.

290. Tanguay, P., Policy responses to drug issues in Malaysia. International Drug Policy Consortium, 2011.

291. Sulaiman, H., Healthcare information Systems Assimilation: The Malaysian Experience. 2011.

292. Istituto nazionale per il Commercio Estero, The pharmaceutical industry in Malaysia 2012.

293. Shazali, N.A., et al., Lean Health Care Practice and Health Care Performance in Malaysian Health Care Industry. International Journal of Scientific and Research Publications, 2013. 3(1).

294. W.H.O., Malaysia Health System Review, in Health Systems in Transition. 2013: Malaysia.

Vietnam

295. Adams, S., Vietnam’s Health Care System: A Macroeconomic Perspective. in International Symposium on Health Care Systems in Asia Hitotsubashi University. 2005. Tokyo.

296. Long, G., Social Health Insurance in Vietnam: Current Issues and Policy Recommendations, in social security extension initiatives in east ASIA S 2007: Tokyo.

297. Business Monitor International, Vietnam Pharmaceuticals & Healthcare Report Q4 2008. 2008.

298. Nguyen, A., et al., Medicine prices, availability, and affordability in Vietnam. Southern Med Review, 2009. 2(2).

299. Respondek, A., Hao, T., Nguyet. N., Vietnam Legal Aspects of the Healthcare System. 2010.

300. Business Monitor International, Vietnam Pharmaceuticals & Healthcare Report Q1 2010. 2010.

301. Nguyen, A., et al., Medicine pricing policies: Lessons from Vietnam. Southern Med Review 2010. 3(2).

302. Nguyen, T., Medicine Prices and Pricing Policies in Vietnam. 2011.

Japan

303. Business Monitor International, Japan Pharmaceuticals & Healthcare Industry Report Q1 2010. 2009.

304. IMS Health., IMS Pharmaceutical Pricing & Reimbursement Concise Guide JAPAN. 2011.

South Korea

305. Chun, C., et al., Health System Review: Republic of Korea, in Health Systems in Transition. 2009.

306. Song, Y., The South Korean Health Care System. JMAJ 2009. 52(3): p. 206-209.

307. Lee, E., The World of HTAs: Review of HTA System in South Korea. in Healthcare Innovation Seminar. 2011. Korea US.

308. IMS Health, IMS Pharmaceutical Pricing & Reimbursement Concise Guide SOUTH KOREA. 2011.

309. Kwon, S., Health Care Reform in Korea: Key Challenges. in IMF Conference. 2011.

310. Yang, B., Drug Pricing and Reimbursement in South Korea. in International HTA Symposium. 2012. U- Tokyo.

311. South Korea World Pharmaceutical MarketEspicom Business Intelligence., Editor. 2012.
