# Supplementary material for: Comprehensive taxonomy and worldwide trends in pharmaceutical policies in relation to country income status
Source: BMC Health Serv Res. 2017 May 25;17:371. doi: 10.1186/s12913-017-2304-2 (PMC5445358; doi:10.1186/s12913-017-2304-2)
Supplement: Supplementary file 2 — Policies applied per country and policy domain. List of the tables with pharmaceutical policies used in each policy domain in the countries considered. (DOCX 62 kb) [file 12913_2017_2304_MOESM2_ESM.docx]

**Appendix B**: Policies applied per country and policy domain

**Table 1.** Direct pricing policies worldwide per type pharmaceutical

|  | ON PATENT | | | | | | | | OFF PATENT | | | | | | | | | | | GENERICS | | | | | | | | | | |  |
| --- | --- | --- | --- | --- | --- | --- | --- | --- | --- | --- | --- | --- | --- | --- | --- | --- | --- | --- | --- | --- | --- | --- | --- | --- | --- | --- | --- | --- | --- | --- | --- |
|  | FP | VBP | CP | CpP | IRP | ERP | PC | HTA | FP | DN | IRP | CpP | ERP | D2 | DP | D3 | D4 | D5 | SC | FP | CpP | IRP | ERP | D2 | D3 | DP | D4 | D5 | D6 | SC |  |
| Austria |  |  |  |  |  | √ |  |  |  |  |  |  |  |  |  |  | √ | √√ |  |  |  |  | √ |  |  | √ |  | √ | √√ |  |  |
| Belgium |  |  |  |  |  |  | √ |  |  |  |  |  |  |  |  |  | √ | √√ |  |  |  |  |  |  |  |  | √ |  |  |  |  |
| Bulgaria |  |  |  |  |  | √ |  |  |  |  |  |  | √ |  |  |  |  |  |  |  |  |  |  |  |  |  | √ |  |  |  |  |
| Cyprus |  |  |  | √ |  | √ | √√ |  |  |  |  |  | √ |  |  |  |  |  | √√ |  | √ |  |  |  | √√ |  |  |  |  |  |  |
| Czech Rep |  |  |  | √ |  | √√ |  | √ |  |  |  |  |  | √ |  |  |  |  |  |  | √ |  | √ |  | √√ |  |  |  |  |  |  |
| Denmark | √ |  |  |  |  |  |  |  | √ |  |  |  |  |  |  |  |  |  |  | √ |  |  |  |  |  |  |  |  |  |  |  |
| Estonia |  | √ |  |  | √ | √ |  |  |  | √√ | √ |  | √ |  |  |  |  |  |  |  | √ |  |  | √ | √ | √√ | √√ | √√ |  |  |  |
| Finland |  | √ |  | √ | √√ | √ |  |  |  |  | √√ |  | √ |  |  |  |  |  |  |  |  |  |  |  |  |  |  | √√ |  |  |  |
| France |  | √√ |  | √ |  |  |  |  |  |  |  |  |  | √ |  |  |  |  |  |  |  |  |  |  |  |  |  |  | √ |  |  |
| Germany | √√ | √ |  |  |  |  |  |  | √ |  |  |  |  |  |  |  |  |  |  | √ |  |  |  |  |  |  |  |  |  |  |  |
| Greece |  |  |  |  |  | √ |  |  |  |  |  |  |  |  |  |  |  | √ |  |  |  |  |  |  |  |  |  |  | √ |  |  |
| Hungary |  | √√ |  |  | √ | √ |  |  |  | √ |  |  | √√ |  |  |  |  |  |  |  |  |  |  |  |  | √ |  | √√ |  | √√ |  |
| Ireland |  |  |  |  |  | √ |  |  |  |  |  |  |  |  | √ |  |  | √√ |  |  |  |  |  |  |  |  |  | √ |  |  |  |
| Italy |  | √√ |  |  | √ | √ |  | √ |  | √ |  |  |  |  |  | √√ |  |  |  |  |  |  |  |  | √ |  |  |  |  |  |  |
| Latvia |  | √√ |  |  | √ | √ |  | √ |  | √√ | √ |  | √ |  |  |  |  |  |  |  | √ | √ | √√ |  |  |  |  |  |  |  |  |
| Lithuania |  | √ |  |  | √ | √√ |  |  |  | √ |  |  | √√ |  |  |  |  |  |  |  |  | √ | √ |  |  |  | √√ |  |  |  |  |
| Luxembourg |  |  |  |  |  | √ |  |  |  |  |  |  | √ |  |  |  |  |  |  |  |  | √ | √√ |  |  |  |  |  |  |  |  |
| Malta | √ |  |  |  |  |  | √√ |  | √ |  |  |  |  |  |  |  |  |  | √ | √ |  |  |  |  |  |  |  |  |  | √√ |  |
| Netherlands |  |  |  |  |  | √ |  |  |  |  |  |  | √ |  |  |  |  |  |  |  |  |  | √ |  |  |  |  |  |  |  |  |
| Poland |  | √√ |  |  | √ | √ |  |  |  | √√ | √ |  | √ |  |  |  |  |  |  |  |  |  |  |  | √ |  |  |  |  |  |  |
| Portugal |  |  |  |  | √ | √√ |  |  |  |  |  | √ | √√ |  |  |  |  |  |  |  |  |  |  |  | √ | √ | √√ |  |  |  |  |
| Romania |  |  |  | √ |  | √√ |  |  |  |  |  |  |  |  |  |  | √ |  |  |  |  |  |  |  |  |  | √ |  |  |  |  |
| Slovakia |  |  |  | √ | √ | √√ |  |  |  |  |  |  | √ |  |  |  |  |  |  |  |  |  |  |  |  |  |  |  | √ |  |  |
| Slovenia |  |  |  |  |  | √ |  |  |  |  |  |  | √ |  |  |  |  |  |  |  |  |  |  | √ |  |  |  |  |  |  |  |
| Spain |  |  |  |  | √ | √√ |  |  |  |  |  |  |  | √ |  |  |  |  |  |  |  |  |  |  |  |  |  | √ |  |  |  |
| Sweden | √ | √ |  |  |  |  |  | √ |  |  |  |  |  |  |  |  |  | √ |  |  |  |  |  |  |  |  |  | √ |  |  |  |
| England | √ |  |  |  |  |  |  |  | √ |  |  |  |  |  |  |  |  |  |  | √ |  |  |  |  |  |  |  |  |  |  |  |
| Croatia |  |  |  |  |  | √ |  |  |  |  |  |  | √√ | √√ |  |  |  |  |  |  |  |  | √√ |  | √√ |  |  |  |  |  |  |
| Norway |  |  |  |  |  | √ |  |  |  |  |  |  |  |  |  |  |  |  | √ |  |  |  |  |  |  |  |  |  |  | √ |  |
| Russia |  |  |  | √ | √ | √√ | √√ |  |  | √ | √ | √ | √ |  |  |  |  |  | √√ |  | √ | √ | √ |  |  |  |  |  |  |  |  |
| Switzerland |  | √ |  |  | √ | √ |  | √ |  |  | √ |  | √ |  |  |  |  |  |  |  |  |  |  |  | √ | √ | √ | √ | √ | √√ |  |
| Turkey |  |  |  | √ | √ | √√ |  |  |  |  |  |  | √√ |  |  |  |  | √√ |  |  |  |  | √ |  |  |  |  | √√ |  |  |  |
| Ukraine |  |  |  |  |  |  | √ |  |  |  |  |  |  |  |  |  |  |  | √ |  |  |  | √ |  |  |  |  |  |  | √√ |  |
| Argentina | √ |  |  |  |  |  | √√ |  | √ |  |  |  |  |  |  |  |  |  | √√ | √ |  |  |  |  |  |  |  |  |  | √ |  |
| Brazil |  |  |  |  | √ | √√ |  | √ |  |  | √ |  | √√ |  |  |  |  |  |  |  |  |  |  |  |  |  | √ |  |  |  |  |
| Chile | √ |  |  |  |  |  | √√ |  | √ |  |  |  |  |  |  |  |  |  | √√ | √ |  |  |  |  |  |  |  |  |  | √√ |  |
| Colombia | √ | √ |  |  | √√ |  |  |  | √ |  |  |  |  |  |  |  |  |  | √√ | √ |  |  |  |  |  |  |  |  |  | √√ |  |
| Mexico | √ | √√ |  |  |  | √ |  | √ |  |  |  |  |  |  |  |  |  |  | √ |  |  |  |  |  |  |  |  |  |  | √ |  |
| Venezuela | √ | √ |  | √ |  |  | √√ |  | √ |  |  |  |  |  |  |  |  |  | √√ | √ |  |  |  |  |  |  |  |  |  | √√ |  |
| Peru |  |  |  |  | √ | √ | √√ |  | √ |  |  |  |  |  |  |  |  |  | √√ | √ |  |  |  |  |  |  |  |  |  | √√ |  |
| Canada | √ | √√ |  |  | √ | √ |  |  | √ |  | √√ |  |  |  |  |  |  |  |  | √ |  |  |  |  | √√ |  |  |  |  |  |  |
| USA | √√ | √√ |  |  |  |  |  |  | √√ | √√ |  |  |  |  |  |  |  |  |  | √√ | √√ |  |  |  |  |  |  |  |  |  |  |
| Egypt |  |  |  | √ |  | √ | √√ | √ |  |  |  |  |  |  |  |  |  |  | √ |  |  |  |  |  |  |  |  |  |  | √ |  |
| Ethiopia |  |  |  |  |  |  | √ |  |  |  |  |  |  |  |  |  |  |  | √ |  |  |  |  |  |  |  |  |  | √ | √√ |  |
| Nigeria | √ |  |  |  |  |  | √√ |  | √ |  |  |  |  |  |  |  |  |  | √ | √ |  |  |  |  |  |  |  |  | √ |  |  |
| South Africa |  |  |  |  | √ | √√ |  |  |  |  |  |  |  |  |  |  |  |  | √ |  |  |  |  |  |  |  |  |  | √ |  |  |
| Saudi Arabia |  |  |  |  |  | √ | √√ | √ |  |  |  |  |  |  |  | √ |  |  |  |  |  |  |  |  |  | √ |  |  | √√ |  |  |
| UAE |  |  |  |  |  | √ |  |  |  |  |  |  | √ |  |  |  |  |  |  |  |  |  |  |  |  |  |  | √ |  |  |  |
| Iran |  |  |  | √ |  | √ | √√ |  |  |  |  | √ | √ |  |  |  |  |  | √√ |  | √ |  |  |  |  |  |  |  |  | √√ |  |
| Israel |  |  |  |  |  | √ |  |  |  |  |  |  | √ |  |  |  |  |  |  |  |  |  |  |  | √ |  |  |  |  |  |  |
| Australia |  | √√ |  | √ | √ | √ |  | √ |  |  |  | √ |  | √√ |  |  |  |  |  |  |  | √ |  | √√ |  |  |  |  |  |  |  |
| Bangladesh |  |  |  |  |  |  | √ |  |  |  |  |  |  |  |  |  |  |  | √ |  |  |  |  |  |  |  |  |  | √ |  |  |
| China |  |  |  | √ | √ | √ | √√ | √ |  |  |  |  |  |  |  |  |  |  | √ |  | √ |  |  |  |  | √ | √ |  | √√ |  |  |
| India |  |  |  |  |  |  | √ |  |  |  |  |  |  |  |  |  |  |  | √ |  |  |  |  |  |  |  |  |  | √ |  |  |
| Indonesia | √ |  |  |  |  |  | √√ |  |  |  |  |  |  |  |  |  |  |  | √ |  |  |  |  |  |  |  |  |  | √ |  |  |
| Japan |  | √√ |  | √ | √ | √ | √ | √ |  |  |  |  |  |  |  |  |  |  | √ |  |  |  |  |  |  |  | √ |  |  | √√ |  |
| Pakistan |  |  |  |  |  | √ | √√ |  |  |  |  |  |  |  |  |  |  |  | √ |  |  |  |  |  |  |  | √ |  | √ | √√ |  |
| Philippines | √ |  |  |  |  | √ | √√ |  | √ |  |  |  | √ |  |  |  |  |  | √ | √ |  |  |  |  |  |  |  |  |  | √√ |  |
| New Zealand |  |  |  |  | √ |  | √ |  |  |  |  |  |  |  |  |  |  |  | √ |  |  |  |  |  |  |  |  |  |  | √ |  |
| South Korea |  | √√ | √ |  |  | √ |  | √ |  |  |  |  |  | √ |  |  |  |  |  |  |  |  |  |  | √ |  |  |  |  |  |  |
| Thailand |  |  |  |  |  |  | √ |  |  |  |  |  |  |  |  |  |  |  | √ |  |  |  |  |  |  |  |  |  |  | √ |  |
| Vietnam | √ |  |  |  |  | √ | √√ |  | √ |  |  | √ |  |  |  |  |  |  | √√ | √ |  |  |  |  |  |  |  |  | √ | √√ |  |
| Malaysia |  |  |  |  |  |  | √ |  |  |  |  |  |  |  |  |  |  |  | √ |  |  |  |  |  |  |  |  |  |  | √ |  |

FP: free pricing and no control; VBP: negotiations and value based pricing; CP:Conditional pricing; CpP: cost plus pricing; IRP: internal reference pricing; ERP: external reference pricing; PC: state price control and setting and tenders; HTA: health technology assesmnet use in pricing; DN: compulasory discounts and negotiations; D2: price discounts up to 20%; D3: price discounts between 20% and 30%; D4: price discounts between 30% and 40%; D5: price discounts between 40% and 50%; D6: price discounts above 50%; SC: State controls.

**Table 2.** Indirect price and cost control policies worldwide

|  | DN | R | PVA | RSA | PB | PC | CB | T | EPC |
| --- | --- | --- | --- | --- | --- | --- | --- | --- | --- |
| Austria | √ | √ | √ |  |  |  |  |  | √ |
| Belgium | √ | √ | √ |  | √ |  | √ |  | √ |
| Bulgaria | √ | √ |  |  |  |  |  |  | √ |
| Cyprus |  |  |  |  |  |  |  | √ | √ |
| Czech Rep |  |  |  |  |  |  |  |  | √ |
| Denmark |  |  |  |  |  |  |  | √ | √ |
| Estonia | √ | √ | √ |  |  |  |  | √ |  |
| Finland |  |  |  |  |  |  |  |  | √ |
| France | √ | √ | √ | √ | √ |  | √ |  | √ |
| Germany | √ | √ | √ | √ |  |  |  | √ | √ |
| Greece | √ | √ |  |  |  |  | √ |  | √ |
| Hungary |  |  | √ | √ | √ |  | √ |  | √ |
| Ireland | √ | √ |  |  |  |  |  | √ | √ |
| Italy | √ |  | √ | √ | √ |  |  |  | √ |
| Latvia |  |  | √ |  |  |  |  | √ | √ |
| Lithuania |  |  |  |  |  |  |  | √ |  |
| Luxembourg | √ |  |  |  |  |  |  |  |  |
| Malta |  |  |  |  |  |  |  | √ |  |
| Netherlands | √ |  | √ |  |  |  | √ | √ | √ |
| Poland | √ | √ | √ | √ | √ |  |  |  | √ |
| Portugal | √ |  | √ |  | √ |  |  | √ | √ |
| Romania | √ |  |  |  |  |  | √ |  |  |
| Slovakia |  |  |  |  |  |  |  |  | √ |
| Slovenia | √ |  |  |  |  |  |  |  |  |
| Spain | √ |  |  |  | √ |  | √ | √ | √ |
| Sweden | √ |  | √ |  |  |  |  |  | √ |
| England | √ |  | √ | √ |  | √ | √ | √ |  |
| Croatia | √ | √ | √ |  | √ |  |  | √ |  |
| Norway |  |  | √ | √ |  |  |  |  |  |
| Russia |  |  |  |  |  |  |  | √ |  |
| Switzerland | √ |  |  |  | √ |  |  |  | √ |
| Turkey | √ |  |  |  |  |  |  |  | √ |
| Ukraine | √ |  |  |  |  |  |  | √ | √ |
| Argentina | √ | √ | √ | √ |  |  |  |  | √ |
| Brazil | √ |  |  |  |  |  |  | √ | √ |
| Chile |  |  |  |  |  |  |  | √ | √ |
| Colombia |  |  |  |  |  |  |  | √ | √ |
| Mexico | √ |  |  |  |  |  |  | √ | √ |
| Venezuela | √ |  |  |  |  |  |  | √ | √ |
| Peru | √ |  |  |  |  |  |  | √ | √ |
| Canada | √ | √ | √ | √ | √ |  | √ |  | √ |
| USA | √ | √ | √ | √ | √ |  | √ |  |  |
| Egypt |  |  |  |  |  |  |  | √ |  |
| Ethiopia |  |  |  |  |  |  |  | √ |  |
| Nigeria |  |  |  |  |  |  |  | √ |  |
| South Africa |  |  |  |  |  |  |  | √ | √ |
| Saudi Arabia |  |  |  |  |  |  |  | √ | √ |
| UAE |  |  | √ | √ |  | √ |  | √ | √ |
| Iran |  |  |  |  |  | √ |  | √ |  |
| Israel | √ |  |  |  |  |  |  |  |  |
| Australia | √ | √ | √ | √ |  |  |  |  | √ |
| Bangladesh |  |  |  |  |  |  |  | √ | √ |
| China | √ |  |  |  |  |  |  | √ | √ |
| India | √ | √ | √ |  |  |  |  | √ | √ |
| Indonesia |  |  |  |  |  |  |  |  | √ |
| Japan | √ |  | √ |  |  |  |  |  | √ |
| Pakistan | √ |  |  |  |  |  |  |  |  |
| Philippines |  |  |  |  |  |  |  |  | √ |
| New Zealand | √ | √ | √ | √ | √ |  |  | √ | √ |
| South Korea |  |  | √ |  | √ |  |  |  | √ |
| Thailand |  |  |  |  |  |  |  |  | √ |
| Vietnam | √ |  |  |  |  |  |  | √ | √ |
| Malaysia |  |  |  |  |  |  |  | √ | √ |

DN: compulasory discounts and negotiations; R: Rebates;

PVA: price volume agreements; RSA: risk sharing agreements;

PB: pay backs; CB: claw back; T: tenders; PC: profit controls;

EPC: extra price cuts, freezes, caps, import mandates.

**Table 3:** Pharmaceutical coverage policies worldwide

|  | BREADTH | SCOPE | | | | | DEPTH | | | | | | | | | |
| --- | --- | --- | --- | --- | --- | --- | --- | --- | --- | --- | --- | --- | --- | --- | --- | --- |
|  | C | VF | EDL | EDL+ | PL | NL | FR | FC | PC | RC | IPR | CL | RP | HTA | PM | D |
| Austria | 5 |  |  |  | √ | √ | √ | √ |  |  |  |  |  | √ | √ |  |
| Belgium | 5 |  |  |  | √ |  |  | √ |  | √ | √ | 5 | Low | √ | √ |  |
| Bulgaria | 3 |  |  | √ |  |  |  |  |  | √ | √ | 45 | Lowest | √ |  |  |
| Cyprus | 5 |  |  | √ |  |  |  |  |  | √ |  |  |  | √ | √ |  |
| Czech Rep | 5 |  |  |  | √ |  |  | √ |  |  | √ | 45 | Lowest | √ | √ |  |
| Denmark | 5 |  |  |  | √ |  |  |  | √ |  | √ | 5 | Lowest | √ | √ | √ |
| Estonia | 5 |  |  |  | √ |  |  | √ |  | √ | √ | 5 | Lowest | √ | √ | √ |
| Finland | 5 |  |  |  | √ |  |  | √ |  | √ | √ | 5 | Lowest | √ | √ | √ |
| France | 5 |  |  |  | √ |  |  | √ |  | √ | √ | 5 | Average | √ | √ |  |
| Germany | 5 |  |  |  |  | √ |  | √ | √ |  | √ | 345 | Low | √ | √ | √ |
| Greece | 5 |  |  |  | √ | √ |  |  |  | √ | √ | 45 | Lowest | √ |  |  |
| Hungary | 5 |  |  |  | √ | √ |  | √ |  | √ | √ | 45 | Lowest | √ | √ |  |
| Ireland | 4 |  |  |  | √ |  | √ | √ | √ |  | √ | 5 | Lowest | √ | √ |  |
| Italy | 5 |  |  |  | √ |  |  | √ | √ |  | √ | 45 | Lowest | | √ |  |
| Latvia | 5 |  |  |  | √ |  |  |  |  | √ | √ | 345 | Lowest | √ |  |  |
| Lithuania | 5 |  |  |  | √ |  |  |  | √ |  | √ | 5 | Lowest | √ |  |  |
| Luxembourg | 5 |  |  |  | √ | √ |  |  |  | √ |  |  |  | √ | √ | √ |
| Malta | 5 |  |  | √ |  |  | √ |  |  |  |  |  |  | √ | √ |  |
| Netherlands | 5 |  |  |  | √ |  |  | √ |  |  | √ | 345 | Average | √ | √ | √ |
| Poland | 5 |  |  |  | √ |  |  | √ |  | √ | √ | 345 | Lowest | √ |  | √ |
| Portugal | 5 |  |  |  | √ |  |  |  |  | √ | √ | 5 | Average | √ |  |  |
| Romania | 5 |  |  |  | √ |  |  | √ |  | √ | √ | 5 | Lowest | √ | √ | √ |
| Slovakia | 5 |  |  |  | √ |  |  | √ |  | √ | √ | 45 | Lowest | √ | √ |  |
| Slovenia | 5 |  |  |  | √ |  |  |  |  | √ | √ | 45 | Lowest | √ | √ | √ |
| Spain | 5 |  |  |  | √ | √ |  |  |  | √ | √ | 5 | Lowest | √ | √ |  |
| Sweden | 5 |  |  |  | √ |  |  |  | √ |  |  |  |  | √ | √ | √ |
| England | 5 |  |  |  |  | √ | √ | √ |  |  |  |  |  | √ | √ |  |
| Croatia | 5 |  | √ | √ |  |  |  | √ |  | √ | √ | 3,4,5 | Lowest | √ | √ |  |
| Norway | 5 |  |  |  | √ |  |  |  | √ |  | √ | 5 | Stepwise | √ | √ | √ |
| Russia | 2 |  | √ | √ |  |  | √ |  | √ |  |  |  |  | √ |  |  |
| Switzerland | 5 |  |  |  | √ |  |  | √ | √ |  |  |  |  | √ | √ | √ |
| Turkey | 5 |  |  |  | √ |  |  |  |  | √ | √ | 5 | Lowest | √ | √ |  |
| Ukraine | 1 |  | √ |  |  |  | √ |  |  |  |  |  |  | √ |  |  |
| Argentina | 5 |  | √ | √ |  |  |  |  |  | √ |  |  |  | √ | √ |  |
| Brazil | 4 |  | √ | √ |  |  | √ |  |  |  |  |  |  |  | √ |  |
| Chile | 4 |  | √ | √ |  |  |  |  |  | √ |  |  |  |  | √ | √ |
| Colombia | 4 |  | √ |  |  |  |  |  | √ |  |  |  |  | √ |  |  |
| Mexico | 4 |  | √ | √ |  |  | √ |  |  |  |  |  |  | √ | √ |  |
| Venezuela | 4 |  | √ |  |  |  |  |  |  | √ |  |  |  |  |  |  |
| Peru | 4 |  | √ |  |  |  | √ | √ | √ |  |  |  |  |  |  |  |
| Canada | 2 |  |  |  | √ |  |  | √ | √ |  | √ | 5 | Lowest | √ | √ | √ |
| USA | 2 |  |  |  | √ |  | √ | √ | √ |  |  |  |  | √ | √ | √ |
| Egypt | 3 |  | √ |  |  |  | √ |  |  |  |  |  |  |  | √ |  |
| Ethiopia | 1 | √ |  |  |  |  |  | √ | √ |  |  |  |  |  |  |  |
| Nigeria | 1 |  | √ |  |  |  | √ |  |  |  |  |  |  |  |  |  |
| South Africa | 5 |  | √ |  |  |  |  | √ |  | √ | √ | 5 | Lowest | | √ |  |
| Saudi Arabia | 4 |  |  |  | √ |  | √ | √ | √ |  |  |  |  | √ |  |  |
| UAE | 3 |  |  |  | √ |  | √ |  |  |  |  |  |  |  |  |  |
| Iran | 5 |  | √ | √ |  |  |  | √ | √ |  |  |  |  |  |  |  |
| Israel | 5 |  |  |  | √ |  |  | √ |  | √ |  |  |  | √ | √ |  |
| Australia | 5 |  |  |  | √ |  |  | √ |  |  | √ |  |  | √ | √ | √ |
| Bangladesh | 3 |  | √ |  |  |  | √ |  |  |  |  |  |  |  |  |  |
| China | 5 |  | √ | √ |  |  |  | √ | √ | √ |  |  |  | √ | √ |  |
| India | 1 |  | √ |  |  |  | √ |  |  |  |  |  |  |  |  |  |
| Indonesia | 3 |  | √ |  |  |  | √ |  |  |  |  |  |  |  | √ |  |
| Japan | 5 |  |  |  | √ |  | √ |  | √ |  |  |  |  | √ | √ | √ |
| Pakistan | 1 |  | √ |  |  |  | √ |  |  |  |  |  |  |  |  |  |
| Philippines | 1 | √ | √ |  |  |  | √ |  |  |  |  |  |  | √ |  |  |
| New Zealand | 5 |  |  |  | √ |  |  | √ |  | √ | √ | 5 | Lowest | | √ | √ |
| South Korea | 5 |  |  |  | √ |  |  |  | √ |  |  |  |  | √ | √ |  |
| Thailand | 4 |  | √ |  |  |  | √ |  | √ |  |  |  |  | √ |  |  |
| Vietnam | 2 |  | √ |  |  |  | √ | √ | √ |  |  |  |  |  | √ |  |
| Malaysia | 4 |  | √ |  |  |  | √ |  |  |  |  |  |  |  |  |  |

C: % of population covered by statutory schemes for pharmaceuticals (0-20%: 1, 20-40%: 2, 40-60%: 3, 60-80%: 4, 80%+: 5); VF: very few drugs covered; EDL: drugs on esential drug list covered; EDL+: additional drugs on top of EDL covered; PL: all drugs on positive list covered; NL: all drugs not on negative list covered; FR: full price reimbursemeht; FC: fixed copayment; PC: percentage copayment; RC: rate category copayment; IPR: internal price referec system in place; CL: level of clusters; RP: refence price determination; HTA: health technology assesment in reimbursement; PM: existence of protection mechanisms; D: deductibles applied.

**Table 4:** Dispensing and demand control policies

|  | DEMAND CONTROL | | | | | | | | | | | | DISPENSING CONTROL | | | | | |
| --- | --- | --- | --- | --- | --- | --- | --- | --- | --- | --- | --- | --- | --- | --- | --- | --- | --- | --- |
|  | ECP | PAT | IPG | IIP | PDM | EMP | CPG | PQT | PPB | SIT | CIP | PRA | NP | SD | ISI | SMP | CS1 | CS2 |
| Austria | √ | √ | √ |  | √ |  | √ | √ |  |  |  | √ |  | √ |  |  |  |  |
| Belgium | √ | √ | √ | √ | √ | √ | √ | √ |  | √ |  | √ |  |  | √ |  |  |  |
| Bulgaria | √ |  | √ | √ |  |  |  |  |  |  |  |  |  |  | √ |  |  |  |
| Cyprus | √ |  | √ | √ |  |  |  |  |  |  |  |  |  | √ | √ | √ | √ |  |
| Czech Rep |  |  | √ | √ |  |  |  |  | √ |  |  | √ | √ |  |  |  |  |  |
| Denmark | √ | √ | √ | √ | √ |  |  |  |  |  |  | √ |  |  |  |  | √ |  |
| Estonia | √ | √ | √ |  | √ | √ | √ |  | √ | √ | √ |  |  |  | √ |  |  |  |
| Finland | √ | √ | √ | √ | √ | √ |  |  |  |  |  |  |  |  |  |  | √ |  |
| France | √ | √ | √ | √ | √ | √ |  | √ |  | √ |  | √ |  |  | √ | √ |  |  |
| Germany | √ | √ | √ | √ | √ | √ | √ | √ | √ | √ |  |  |  |  |  |  | √ |  |
| Greece | √ |  | √ |  | √ | √ |  | √ |  |  | √ |  |  |  |  |  |  | √ |
| Hungary | √ | √ | √ | √ | √ | √ | √ |  | √ | √ |  | √ |  |  | √ |  |  |  |
| Ireland | √ | √ | √ |  | √ |  |  |  | √ |  | √ |  |  |  |  |  |  | √ |
| Italy | √ | √ | √ |  | √ | √ | √ | √ | √ | √ | √ | √ |  |  |  |  |  | √ |
| Latvia | √ |  | √ | √ | √ |  |  |  | √ |  |  | √ |  |  | √ |  |  |  |
| Lithuania |  | √ | √ |  | √ | √ |  |  | √ | √ | √ |  |  |  |  | √ |  |  |
| Luxembourg | √ | √ | √ | √ | √ |  |  |  |  |  |  |  |  |  | √ |  |  |  |
| Malta |  |  |  | √ |  |  |  |  |  |  |  |  |  |  |  |  |  | √ |
| Netherlands | √ | √ | √ | √ | √ | √ | √ | √ |  | √ |  | √ |  |  |  | √ |  |  |
| Poland | √ | √ | √ | √ | √ |  |  | √ |  |  |  |  |  |  | √ |  |  |  |
| Portugal | √ | √ | √ |  | √ | √ |  |  |  | √ | √ |  |  |  |  |  | √ |  |
| Romania | √ | √ | √ |  | √ | √ |  | √ |  |  | √ |  |  |  |  |  |  | √ |
| Slovakia | √ | √ | √ | √ | √ | √ | √ | √ | √ |  |  |  |  |  |  |  |  | √ |
| Slovenia | √ | √ | √ | √ | √ |  |  |  |  | √ |  |  |  |  | √ | √ |  |  |
| Spain | √ | √ | √ |  | √ | √ | √ | √ |  | √ | √ | √ |  |  |  |  |  | √ |
| Sweden | √ | √ | √ | √ | √ | √ | √ | √ | √ | √ |  |  |  |  |  | √ |  | √ |
| England | √ | √ | √ | √ | √ | √ | √ | √ | √ | √ |  |  |  |  | √ | √ |  |  |
| Croatia | √ |  | √ |  | √ |  |  | √ |  |  |  |  |  |  | √ |  |  |  |
| Norway | √ | √ | √ | √ | √ | √ |  | √ |  |  |  |  |  |  | √ | √ |  |  |
| Russia |  | √ | √ | √ | √ |  |  |  |  |  |  |  |  |  | √ |  |  |  |
| Switzerland |  | √ | √ |  | √ | √ |  |  |  | √ |  |  |  |  | √ | √ |  |  |
| Turkey |  | √ | √ | √ | √ | √ |  |  |  |  |  |  |  |  | √ |  |  |  |
| Ukraine | √ |  | √ |  |  |  |  |  |  |  |  |  |  |  | √ |  |  |  |
| Argentina | √ |  | √ | √ | √ |  |  |  |  |  | √ |  |  |  | √ |  |  |  |
| Brazil |  |  | √ | √ |  |  |  |  |  |  |  |  |  |  | √ |  |  |  |
| Chile |  |  |  |  |  |  |  |  |  |  | √ |  |  |  | √ |  |  |  |
| Colombia |  |  | √ | √ |  |  |  |  |  |  |  |  |  |  | √ |  |  |  |
| Mexico |  |  | √ |  | √ | √ |  |  |  | √ | √ |  |  |  | √ |  |  |  |
| Venezuela |  |  |  | √ |  |  |  |  |  |  |  |  |  |  | √ | √ |  |  |
| Peru | √ |  |  |  |  |  |  |  |  |  | √ |  |  |  | √ |  |  |  |
| Canada | √ | √ | √ | √ | √ | √ |  | √ |  |  |  |  |  |  |  |  |  | √ |
| USA | √ | √ | √ | √ | √ | √ | √ | √ | √ |  |  | √ |  |  | √ | √ | √ |  |
| Egypt | √ |  | √ |  |  |  |  |  |  | √ |  |  |  |  | √ |  |  |  |
| Ethiopia | √ |  |  |  |  |  |  |  |  |  |  |  |  |  | √ |  |  |  |
| Nigeria | √ |  |  |  |  |  |  |  |  |  |  |  |  |  | √ |  |  |  |
| South Africa | √ | √ | √ | √ | √ |  |  |  | √ |  |  |  |  |  |  |  |  | √ |
| Saudi Arabia | √ | √ | √ |  |  |  |  |  |  |  |  |  | √ |  |  |  |  |  |
| UAE |  | √ | √ |  |  |  |  |  |  |  |  |  | √ |  |  |  |  |  |
| Iran | √ | √ | √ | √ | √ |  |  |  |  |  |  |  | √ |  |  |  |  |  |
| Israel | √ | √ |  |  | √ |  |  |  |  |  |  |  |  |  | √ |  |  |  |
| Australia | √ | √ | √ | √ | √ | √ |  |  |  |  |  |  |  |  | √ | √ |  |  |
| Bangladesh | √ |  |  |  |  |  |  |  |  |  |  |  | √ |  |  |  |  |  |
| China | √ | √ |  | √ |  |  |  |  |  |  |  |  |  |  | √ |  |  |  |
| India | √ |  |  | √ |  |  |  |  |  |  |  |  | √ |  |  |  |  |  |
| Indonesia | √ |  | √ |  |  |  |  |  |  |  |  |  | √ |  |  |  |  |  |
| Japan | √ | √ | √ | √ | √ | √ |  |  |  |  |  |  |  |  | √ | √ |  |  |
| Pakistan | √ |  |  |  |  |  |  |  |  |  |  |  | √ |  |  |  |  |  |
| Philippines | √ |  |  |  |  |  |  |  |  | √ |  |  |  |  | √ |  |  |  |
| New Zealand | √ | √ | √ | √ | √ |  |  | √ | √ | √ |  |  |  |  | √ |  |  |  |
| South Korea | √ | √ | √ | √ | √ | √ |  |  |  | √ |  |  |  |  | √ | √ |  |  |
| Thailand | √ |  |  |  |  |  |  |  |  |  |  |  |  |  | √ |  |  |  |
| Vietnam | √ |  | √ |  |  |  |  |  |  |  |  |  |  |  | √ |  |  |  |
| Malaysia | √ |  |  |  |  |  |  |  |  |  |  |  | √ |  |  |  |  |  |

ECP: Educational campaigns and programs; PAT: prescription aiding tools; IPG indicative prescription guidelines; IIP: indicative INN prescription; PDM: monitoring of prescribing and prescription data; EMP: electronic mandatory prescription; CPG: compulsory prescription guidelines; PQT: prescription quotas and targets; PPB: predefined doctor prescription budgets; SIT: Sanctions and incentives for target/guideline adherence; CIP: compulsory INN prescription; PRA: prior and posterior restrictions/approvals; NP: no strong policies in place; SD: subsitution disallowed; ISI: indicative substitution and incentives; SMP: strong measures for substitution; CS1: compulsory substitution from range of products; CS2: compulsory substitution from the cheapest.

**Table 5:** Countries by income group

| High income | Middle income | Low income |
| --- | --- | --- |
| Australia | Argentina | Bangladesh |
| Austria | Bulgaria | Egypt |
| Belgium | Brazil | Ethiopia |
| Canada | China | India |
| Chile | Colombia | Indonesia |
| Croatia | Hungary | Nigeria |
| Cyprus | Iran | Pakistan |
| Czech Republic | Malaysia | Philippines |
| Denmark | Mexico | Ukraine |
| Estonia | Peru | Vietnam |
| Finland | Romania |  |
| France | South Africa |  |
| Germany | Turkey |  |
| Greece | Thailand |  |
| Israel | Venezuela |  |
| Ireland |  |  |
| Italy |  |  |
| Japan |  |  |
| Latvia |  |  |
| Lithuania |  |  |
| Luxembourg |  |  |
| Malta |  |  |
| Netherlands |  |  |
| New Zealand |  |  |
| Norway |  |  |
| Poland |  |  |
| Portugal |  |  |
| Russia |  |  |
| Slovakia |  |  |
| Slovenia |  |  |
| Soudi Arabia |  |  |
| Spain South Korea |  |  |
| Sweden |  |  |
| Switzerland |  |  |
| UAE |  |  |
| United Kingdom |  |  |
| USA |  |  |
